# Must analysis of meaning follow analysis of form? A time course analysis

**DOI:** 10.3389/fnhum.2015.00111

**Published:** 2015-03-11

**Authors:** Laurie B. Feldman, Petar Milin, Kit W. Cho, Fermín Moscoso del Prado Martín, Patrick A. O’Connor

**Affiliations:** ^1^Department of Psychology, University at Albany, State University of New YorkAlbany, NY, USA; ^2^Haskins LaboratoriesNew Haven, CT, USA; ^3^Quantitative Linguistics Group of Harald Baayen, Eberhard Karls UniversityTbingen, Germany; ^4^Faculty of Philosophy, University of Novi SadNovi Sad, Serbia; ^5^Department of Linguistics, University of California, Santa BarbaraSanta Barbara, CA, USA

**Keywords:** reading proficiency, morphological processing, semantic transparency

## Abstract

Many models of word recognition assume *that processing proceeds sequentially from* analysis of form to analysis of meaning. In the context of morphological processing, this implies that morphemes are processed as units of form prior to any influence of their meanings. Some interpret the apparent absence of differences in recognition latencies to targets (SNEAK) in form and semantically similar (sneaky-SNEAK) and in form similar and semantically dissimilar (sneaker-SNEAK) prime contexts at a stimulus onset asynchrony (SOA) of 48 ms as consistent with this claim. To determine the time course over which degree of semantic similarity between morphologically structured primes and their targets influences recognition in the forward masked priming variant of the lexical decision paradigm, we compared facilitation for the same targets after semantically similar and dissimilar primes across a range of SOAs (34–100 ms). The effect of shared semantics on recognition latency increased linearly with SOA when long SOAs were intermixed (Experiments 1A and 1B) and latencies were significantly faster after semantically similar than dissimilar primes at homogeneous SOAs of 48 ms (Experiment 2) and 34 ms (Experiment 3). Results limit the scope of form-then-semantics models of recognition and demonstrate that semantics influences even the very early stages of recognition. Finally, once general performance across trials has been accounted for, we fail to provide evidence for individual differences in morphological processing that can be linked to measures of reading proficiency.

## Introduction

Models of visual word recognition typically assume that some information about the form of a word must be available before access to the word’s meaning is possible. In the absence of any additional knowledge about the word to be recognized, this assumption seems logical. Therefore, when applied to the domain of morphological processing, one might argue that a morpheme is processed as a unit of form prior to any influence of its meaning. This stronger claim is controversial because the classical linguistic position is that morphemes are both, units of form and units of meaning. This contradiction is therefore worthy of further investigation.

Words that share a base morpheme (e.g., SHARP) tend to be similar in meaning as well as form (SHARPER, SHARPLY, SHARPEN, SHARPENER). Generally, however, words that look alike are not necessarily related in meaning (SHARK, SHARE, SHARD, HARP, TARP), and words that have similar meanings do not look alike (ACUTE, ASTUTE, CRISP, DISTINCT, CUNNING, INTELLIGENT). Therefore, morphologically related words represent a partial exception to the general claim that in language, form and meaning are related in a complex, and seemingly arbitrary fashion. Yet many would agree that, in the domain of word recognition, the meaning of a word can be informative about that word’s form and vice versa (see for example, Bybee, 1985). Thus, in the broadest sense, meaning should provide a source of contextual information that could reduce uncertainty in early processing of form.

A framework for word reading and for morphological processing in particular, with an initial stage devoted to the orthographic properties of the input while remaining stubbornly independent of meaning, unnecessarily deprives that stage of a potentially useful source of information. In the present study we document how meaning and form interact continually when processing morphologically complex words, beginning with the earliest registration of input.

### Does Analysis of Meaning Follow Analysis of Form?

Among priming variants of the lexical decision paradigm, briefly presented primes [stimulus onset asynchronies (SOAs) <60 ms] preceded by pattern masks are assumed to capture an early phase of processing (Forster et al., 2003). Under these conditions, similarity between prime and target benefits recognition as evidenced by reduced target decision latencies for similar pairs relative to unrelated controls (facilitation). According to a form-based account of early processing target decision latencies should be faster both for prime-target pairs like sneaky-SNEAK or farmer-FARM (orthographically and semantically similar, often referred to as transparent), and for pairs like sneaker-SNEAK or corner-CORN (orthographically similar, semantically dissimilar, often referred to as opaque) relative to unrelated controls. Crucially, both types of related pairs should be equivalent because they are equally similar in form and semantics plays no role.

Previous studies using a masked priming manipulation report statistically equivalent facilitation for true (prefixed or suffixed) morphological derivations, and for primes that appear to be morphologically complex words but are not (-ER occurs as a suffix in English words such as FARMER but is a pseudosuffix in the word CORNER). Many studies have reported “morphological” facilitation that does not vary reliably with semantic similarity within a prime-target pair (Longtin et al., 2003; Rastle et al., 2004)^1^. Under the same conditions, it is difficult to document facilitation for word pairs like CORNEA-corn or BROTHEL-broth where the prime does not end in a sequence of letters that can function as a suffix (e.g., Rastle et al., 2000, 2004; Longtin et al., 2003; McCormick et al., 2009). Similarly, for non-word primes, facilitation following morphologically structured but not non-suffixed primes (Longtin and Meunier, 2005) is consistent with this account (but see Beyersmann et al., 2014), who showed facilitation following French non-suffixed (flexint-FLEX) as well as suffixed primes (flexent-FLEX) that was comparable in high-proficiency readers. Collectively, facilitation with pairs that appear to be morphologically structured like CORNER-corn provides the foundation for the claim that morphological facilitation in early visual word recognition is based only orthographic structure and the potential to fully decompose a word and isolate its stem, without regard to the semantics of its morphemes. Complementarily, the absence of facilitation for pairs that are only partially decomposable (CORNEA-corn) serves as the foundation for the claim that the effect is morphological and not based only on similar orthographic form in prime and target.

The failure to find a difference (null effect) in magnitudes of facilitation for semantically similar and dissimilar pairs, like SNEAKY-sneak vs. SNEAKER-sneak, in individual experiments provides the foundation for the form-then-meaning account. Within this framework, the potential for successful decomposition determines morphological facilitation and semantic contributions do not arise until a later semantically informed stage that typically requires longer exposure durations of the prime (Feldman, 2000; Rastle et al., 2000; Taft and Kougious, 2004; Meunier and Longtin, 2007; Taft and Nguyen-Hoan, 2010). Accordingly, when semantic contributions are detected in tasks that purportedly tap early processing, they are attributed to feedback activation based on similarity at a morpho-semantic level that accrues fast enough to influence performance in a task that depends on an earlier phase of processing (e.g., Diependaele et al., 2005; Holcomb and Grainger, 2006; Morris et al., 2008, 2011, 2013). This account of semantic effects, however, differs from a ‘supralexical’ account (e.g., Giraudo and Grainger, 2001), where properties of morphemic constituents only become influential after activation of the full word. Finally, both accounts differ from those whose core claim is that form and meaning processes mutually shape each other. Whether one detects evidence of full word or of constituent processing depends on properties of the word that appears and its attributes and they tend to interact in a complex and non-linear manner (e.g., Kuperman et al., 2009).

In models of lexical access, statistically comparable magnitudes of facilitation for semantically similar and dissimilar pairs in individual experiments is taken as primary support for an early morpho-orthographic stage during which semantics play no role (for a review see Rueckl and Aicher, 2008), although a meta-analytic review of the magnitudes of facilitation reveals an early semantic influence (Feldman et al., 2009). Across those studies that were proffered in support of the claim for a semantically blind process (Rastle and Davis, 2008) we reported that facilitation was significantly greater (10 ms) after semantically similar (transparent) than semantically dissimilar (opaque) morphologically related primes. This outcome attests to the role of semantics in a task that captures early processing where morphemes are purported to function as units of form (Feldman et al., 2009 but see Davis and Rastle, 2010). Further, meta-analysis contrasting semantically similar and semantically dissimilar primes demonstrates the risk of interpreting individual (null) findings. In our case it is the claim that parsability of a word’s orthographic structure into stem (SNEAK) and potential affix (Y; ER) proceeds devoid of information about morpho-semantic structure that we contest^2^.

### Concurrent Access to the Semantic and Form Properties of Words in the Neurocognitive System

Challenges to the form-then-meaning assumption of processing are not limited to morphological models in word recognition tasks. Findings of near simultaneous access to the ortho-phonological and semantic properties of whole words are central to some current neurophysiological theories of lexical processing. For instance, Pulvermüller (2002) report that when processing a word, the cortical subnetworks that code semantics rapidly fire when the subnetworks that encode orthographic and/or phonological forms of the words are activated. In Pulvermüller (2002) view, the orthographic and semantic subnetworks form a single functional unit (i.e., a cell assembly in the Hebbian sense). In essence, concurrent access to the semantic and form properties of words seems not to be a peculiarity of masked priming in the lexical decision paradigm. Rather, it seems to be a general property of the neurocognitive system.

Analogous to the behavioral measures, primes with morphological or form similarity to the target typically show negative ERP amplitude in the latency range of 250 ms (N250) or 400 ms (N400) after target onset that is attenuated relative to an unrelated baseline condition (for a review of EEG findings see Smolka et al., 2015). Those ERP studies that report similar patterns for form similar, morphologically structured pairs, with and without semantic similarity, have been marshaled as evidence for a purely orthographic analysis of morphemes that operates at an early stage of visual word recognition. Revealingly, under these conditions relative to an unrelated prime-target pair, form and semantically similar pairs like FARMER-FARM typically generate either an N250 or both N250 and N400 attenuations (cf. Holcomb and Grainger, 2006; Lavric et al., 2007, 2012; Morris et al., 2007, 2008, 2011, 2013). By comparison, form similar and semantically unrelated albeit morphologically structured pairs like CORNER-CORN, and form similar but only partially structured pairs like CORNEA-CORN show less consistent results: no effect for either type, N250 attenuations for both types, or N250 concomitant with N400 attenuations in both types or only in the partially structured pairs (Holcomb and Grainger, 2006; Morris et al., 2007, 2008, 2011, 2013; Lavric et al., 2012).

At a minimum both the meta-analysis of the magnitudes of facilitation based on target decision time after semantically similar and semantically dissimilar morphologically related primes, as well as inconsistencies across ERP studies, highlight the risk of recruiting individual (null) findings as a justification for assigning form and meaning processes to distinct stages.

### Modeling the Time Course Over Which Form and Meaning Interact in Word Recognition

Several studies have investigated the role of a morpheme’s semantic properties by holding form constant while manipulating the semantic similarity of a prime and target that share their base morpheme and then examining patterns of facilitation across long and short SOAs in the lexical decision task (Feldman and Soltano, 1999; Rastle et al., 2000, 2004; Feldman et al., 2002; Longtin et al., 2003; Diependaele et al., 2011). Most individual studies failed to observe reliable effects of semantics precisely when primes were masked and appeared at SOAs shorter than 60 ms^3^. However, one limitation in almost all of those studies was that different targets appeared with form-similar primes that did and did not preserve semantic similarity with the target. Consequently, differences between targets were confounded with semantic transparency. Nonetheless, a pattern begins to emerge suggesting that SOA may play a critical role in the detection of early semantic effects among morphological relatives. In Dutch, Diependaele et al. (2005) demonstrated a different time course for effects of semantically similar and dissimilar primes that were similar in form. Likewise in French with an incremental priming technique (Jacobs et al., 1995), facilitation arose with semantically and form similar primes at 40 ms while facilitation after semantically and form dissimilar primes was first evident only at a 67 ms prime duration. Typically, those manipulations of SOA are between experimental blocks (and often between subjects). Obviously, one can obtain a more detailed characterization of the time course of various types of facilitation if the SOA manipulation is within subjects, items, and experimental blocks. More specifically with these constraints, a joint analysis of responses across the different SOAs and prime types within a single regression model permits a more direct assessment and augments the potential to detect different time courses.

A systematic comparison of facilitation across semantically similar (transparent) and dissimilar (opaque) prime types and across SOAs of 100 ms and shorter, while holding form similarity constant, is the primary objective of the current study. The rational for sampling over a somewhat extended range of SOAs was to enhance interpolation by allowing for more precise diagnostics of possible non-linearities in patterns of facilitation^4^. In this regard, what we see as the main limitation of the studies enumerated above is that each considered at most two SOAs in the range before facilitation transitions from subliminal to conscious. Therefore, on the basis of those restricted data it is not possible to specify the time course over which facilitation emerges and/or disappears, or to differentiate linear from non-linear patterns within the semantic transparency by SOA interaction. The current design thus maximizes the potential to observe a non-linear relationship between SOA and facilitation due to semantic similarity between prime and target.

**FIGURE 1:**
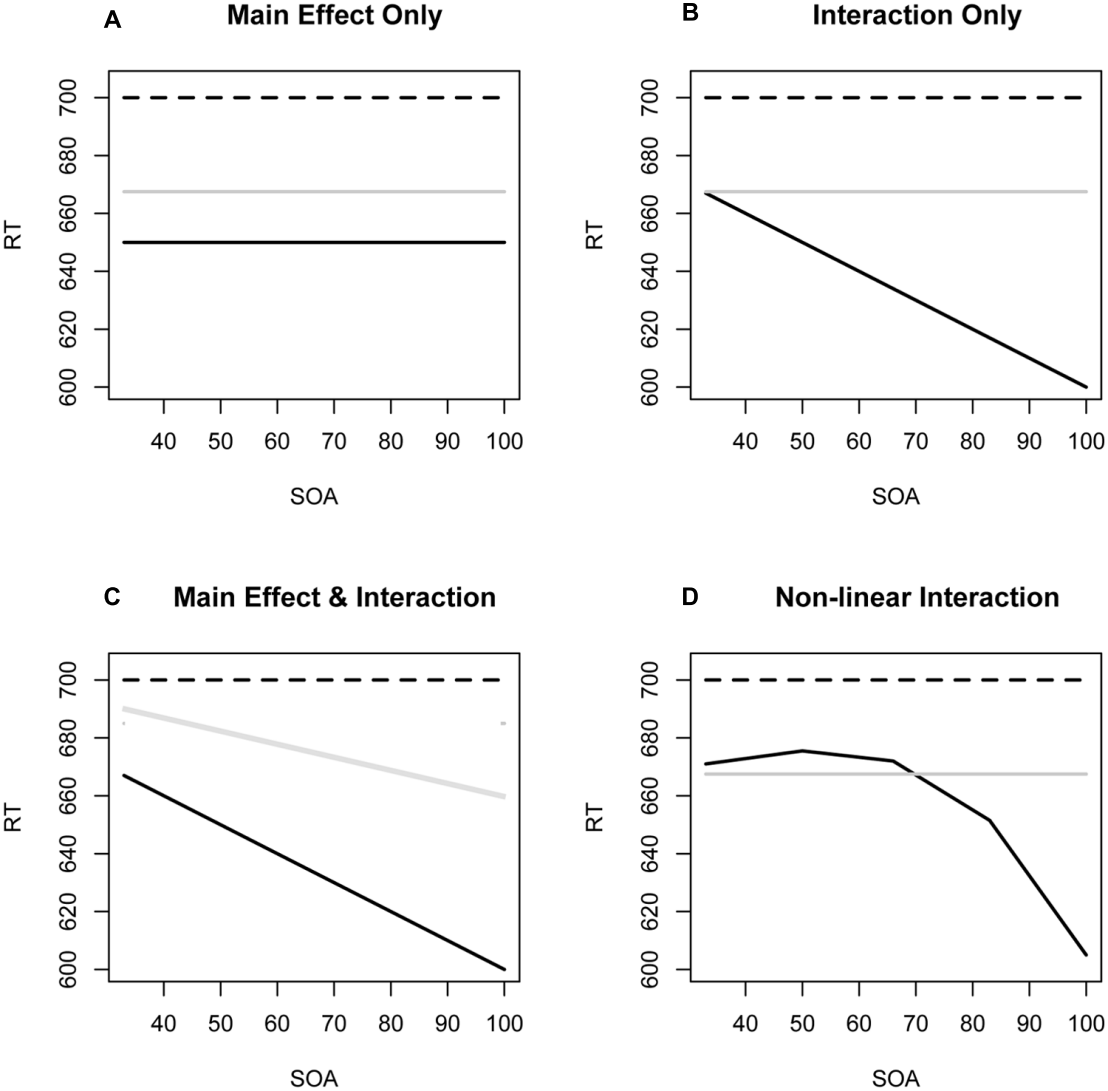
Four theoretically plausible profiles of the evolution of facilitation at very short stimulus onset asynchronies (SOAs) for semantically similar (black solid lines) and semantically dissimilar (gray solid lines) morphologically related pairs relative to unrelated pairs (discontinuous lines). **(A)** Full and instantaneous access to meaning as well as form with no change over SOA. **(B)** Effects of meaning that increase as SOA increases. **(C)** Main effect of form and an effect of meaning that is present at the onset but increases over time. **(D)** Effect of meaning that emerges only after form processing begins.

**Figure 1** illustrates four hypothetical but theoretically plausible profiles of the emergence of facilitation for semantically similar (black solid lines) and semantically dissimilar (gray solid lines) morphologically related pairs, in relation to unrelated pairs (dashed lines). Pattern (A) corresponds to an unlikely “full and instantaneous access” to the meaning as well as the form attributes of a lexical representation in the tradition of opening a lexical entry (Forster et al., 2003). Pattern (B) is a cascaded version of a sequential model where morphological effects are initially form-based and independent of semantics, with a gradually increasing semantic contribution. Within the form then meaning framework where the underlying assumption is that words are decomposed and stems are initially processed in isolation and independently of their morphological context, accounts of semantic contributions sometimes introduce feedback activation based on similarity at a morpho-semantic level that emerges quickly enough to alter early processing. A similar solution has been proposed for form similar pairs with (e.g., Diependaele et al., 2005; Morris et al., 2008) and without (Hino et al., 2002; Pexman et al., 2008) shared morphology. The implication is that semantic effects are not evident at the earliest point at which visual input has been processed, and must await cascading or feedback activation from later in the processing hierarchy. Pattern (C) which we promote, depicts early access to both formal and semantic properties of the word, with a wider semantic neural assembly becoming progressively more activated, in line with the theoretical proposal of Pulvermüller (1999, 2001), Plaut and Gonnerman (2000), and Moscoso del Prado Martín (2007). Here, semantically unrelated pairs that are fully decomposable like BROTHER-broth or CORNER-corn are more similar to pairs that are only partially decomposable like BROTHEL-broth or CORNEA-corn than to semantically similar pairs like BROTHY-broth or FARMER-farm (Milin et al., 2015). The implication is that effects of form and of semantic similarity operate concurrently and interdependently and that contributions increase even across very short SOAs in the 34–67 ms range. Pattern (D) represents the prediction of a purely sequential model in which access to semantic properties is blocked until some basic morphological processing, dependent only on word form, has been completed. This corresponds to models that posit an early morpho-orthographic segmentation stage that remains semantically blind and devoid of cascading semantics for some period of time, such as some readings of Rastle et al. (2004). It posits a discontinuity between discrete stages and thus fails to anticipate graded contributions of meaning. Note that all four patterns assume that decision latencies for the unrelated and semantically dissimilar condition remain relatively unchanged across SOAs shorter than 100 ms. Another version of (C), with no main effect of prime type, could result in a cross-over interaction by which an effect alternates between inhibition and facilitation. Finally, an alternative shape of (D) could show that the difference in similar and dissimilar facilitation is significant only in a particular band of SOA values, and not significant (above or) below that range.

Our goal in the present study is to document semantic influences in the early stages of morphological processing, searching as early as 34 ms. To obtain a more fine-grained characterization of the time-course of activation of the target by the prime, we examine facilitation patterns across a range of SOA values. Experiment 1A includes the three different SOAs of 34, 67, and 84 ms. Experiment 1B includes the SOAs of 48 and 100 ms. All SOAs are short enough to escape strategic processing (Neely, 1991) but vary enough to optimize detection of non-linear patterns of facilitation. Experiment 2 examines the single SOA of 48 ms presented to participants in combination with other SOAs and as a solo SOA. Experiment 3 focuses on a single SOA of 34 ms with consideration of individual differences and their relation to reading skill.

In each experiment, we compare facilitation after semantically similar and dissimilar primes that are forward masked, when both types of primes are highly similar in form to the same target. In earlier studies that have assessed early effects of semantics, different targets appeared with similar primes and with dissimilar primes. Although sets of targets were rigorously matched in those studies, unrelated decision latencies were slower for targets whose related prime context was semantically dissimilar as compared to similar. For example, in Feldman et al. (2009), target latencies (error rates) in the unrelated condition were 20 ms longer (2.8% greater) for dissimilar pairs like corner-CORN than for similar pairs like FARMER-FARM, and in Rastle et al. (2004) that difference was 23 ms (6.1%). Different unrelated baselines make it difficult to determine whether magnitudes of facilitation are comparable across targets whose related primes differ on semantic but not orthographic similarity. In the present study, because the same targets appeared with semantically dissimilar (SNEAKER-sneak) and similar (SNEAKY-sneak) primes we could eliminate any confounding between transparency and facilitation based on target attributes.

## Experiments 1A,B

To obtain a more fine-grained characterization of the time-course of activation of the same targets by different primes, we examine the effect of semantic transparency across five SOA values presented randomly within blocks of trials. This includes an SOA of 48 ms, the conventional duration at which to examine facilitation when primes are forward masked. Experiment 1A includes the three SOAs of 34, 67, and 84 ms. Experiment 1B includes the SOAs of 48 and 100 ms. We also incorporate a principal component analysis (PCA), and then used PC scores as uncorrelated (orthogonal) predictors to offset differences between targets on classical measures of word recognition such as neighborhood size and frequency. Our primary focus is on violations of form-then-meaning processing as revealed by the time course over which evidence of early semantic processing emerges.

### Method

#### Participants

One hundred and eight undergraduates participated in Experiment 1A and 86 in Experiment 1B. All were monolingual students at the University at Albany, and participated in partial fulfillment of the introductory psychology course requirements.

#### Materials

Sixty-three stems were selected as critical word targets. Each appeared with a derivationally related or compound prime^5^. Three primes were created for each target word, and in a given experimental list, a unique third of the items was each paired with semantically similar primes, dissimilar primes, or unrelated primes. The latter were formed from a different stem than their target. In the semantically similar condition, the meaning of the target (e.g., SNEAK, CAB) was retained in the prime (e.g., SNEAKY, CABSTAND). In the semantically dissimilar condition, primes (e.g., SNEAKER, CABBAGE) failed to retain the full meaning of the stem. The dissimilar condition included both semantically opaque primes that were related etymologically to the target (e.g., SNEAKER-SNEAK) as well as pseudo-morphemic relatives (e.g., RATIFY-RAT). Unrelated primes (e.g., KEENEST, HEADSTAND) retained the final letter sequence (EST, STAND) of one of the related primes and had minimal letter overlap^6^. Five semantically similar primes, five semantically dissimilar primes and six unrelated primes were compounds.

The semantically similar and semantically dissimilar primes were closely matched on variables known to influence lexical decision latencies as well as normed single word lexical decision reaction time (Balota et al., 2007). These include length, logged Usenet frequencies in the HAL system (Lund and Burgess, 1996), orthographic neighborhood size, and phonological neighborhood size. In addition, similar and dissimilar primes did not differ on the number of sound, spelling, and sound plus spelling changes from prime to target. Critical stems recurred in full in (complex or compound) prime and in target (FIGLET-FIG; FIGMENT-FIG VS. ARCHWAY-ARCH; ARCHER-ARCH) on 75% of trials. For most pairs, the stem’s spelling and pronunciation were retained fully in the prime (Widmann and Morris, 2009). Exceptions included final e deletion before some suffixes (SLIMY-SLIME) as well as other less systematic changes (PROVEN-PROOF; CELERY-CELL). Most important for our purposes, the number of instances of systematic and unsystematic mismatch was equalized across semantically similar and dissimilar prime types.

**Table 1** summarizes means and SD for attributes of the 63 items that were included in the experiment. Five target items were eliminated from the dataset before analysis. This included two items whose primes were rated as similar, contrary to our initial classification (ABSENT, SEED). Another three items were removed to retain equal number of items per prime condition in the multiple SOA experiments (PIG, FILL, SKIN). They were removed after the 48 ms SOA experiment.

**Table 1:** Attributes of targets and their primes.

		Len	log HAL	ON	PN	Fam	LSA	Rating
Primes	Sem similar	7.0	2.632	2	4	1.5	2.8	5.7
	Sem dissimilar	7.0	2.860	1	4	1.6	0.7	2.7
	Unrelated	7.0	2.608	2	3	1.5		
Targets		4.5	3.977	9	18	5.8		

*LEN, length in letters; log HAL, log HAL frequency; ON, orthographic neighbors; PN, phonological neighbors; FAM, morphological family size; LSA, latent semantic analysis; Rating, prime-target similarity rating*.

Latent semantic analysis cosine values (Landauer et al., 1998) that capture semantic similarity based on the extent to which words appear in the same context and rating judgments based on a 7-point scale indicated that the meaning overlap between prime and target was always higher for semantically similar than for dissimilar pairs^7^. The LSA cosine values (SD) for semantically dissimilar [0.07 (0.20)] and similar [0.28(0.09)] items were significantly different.

As in Feldman et al. (2009), we introduced many ID filler trials and concomitant list-wise semantic similarity so as to maximize evidence of morphological processing and the potential to detect an interaction with semantic transparency in the forward masked primed lexical decision task. (See Appendix A). Experimental lists with a high proportion of lexically identical (ID) prime-target filler trials (e.g., CRACKER–CRACKER) show semantic facilitation even when primes are forward masked and the SOA is brief (Bodner and Masson, 2003). Moreover, the inclusion of form-similar word–word ID and word–non-word quasi-ID trials to create a relatedness proportion of 75% significantly boosts semantic and morphological but not orthographic facilitation (Feldman and Basnight-Brown, 2008).

#### Design

Across participants, all targets were preceded by semantically similar, dissimilar, and unrelated primes equally often. No target was repeated within a session. In Experiment 1A each participant responded to seven pairs in each condition created by the 3 prime type × 3 SOA design. In Experiment 1B each participant responded to 10 pairs in each condition created by the 3 prime type × 2 SOA design. Stimuli were counterbalanced such that across participants, all targets were presented with each prime approximately equally often, and no target was presented more than once to a participant.

In addition to the 63 critical items described above, 42 word–word pairs were included as filler stimuli. All of the word–word filler pairs had identical primes and targets (i.e., “identity” trials). Half of these were morphologically simple words. About one third included an affix and thus were complex. About one sixth were compounds. Each participant responded to 105 word target trials in total. In order to make the relation between high form overlap and target lexicality uninformative (cf., Rastle et al., 2004), 84 of the 105 word–non-word pairs contained the non-word target’s form plus a frequent letter sequence as the ending (e.g., FRUGAL-FRUG) and 21 shared no letters in the same position (YEARBOOK-ANNON).

#### Procedure

Each trial began with a 500 ms fixation mark (+) that appeared in the middle of the screen. An ISI of 48 ms occurred before the forward mask (number of # signs matched to prime length) that lasted 450 ms. The prime then appeared in lowercase letters 34- 67- 84 ms (Experiment 1A) or 48–100 (Experiment 1B) ms and replaced the mask. The target was printed in uppercase letters and replaced the prime in the same position. Targets were visible for 3000 ms or until the participant made a response. The intertrial interval was 1000 ms. There was no mention of the primes in the instructions.

Items were presented in black 16-point font on a white background with E-Prime 2.0 (Psychology Software Tools, Inc.) on a PC-compatible computer with a dell 17 inch LCD, with a 60 Hz refresh rate. A different random order of prime-target pairs appeared for each participant. Participants made a lexical decision for each target by pressing the M key for words and the C key for non-words with their right and left index fingers, respectively. Participants responded to 12 practice trials before the experimental session, and the makeup of the practice stimuli mirrored that of the stimuli in the main experiment. The study was approved by the Institutional Review Board of the University at Albany, State University of New York.

### Results and Discussion

Arithmetic means for prime type across the range of SOAs (34, 48, 67, 84, 100) are summarized in **Table 2**. For the analyses, correct latencies were transformed into their negative reciprocal (-1000/RT), to better approximate normality and homoscedasticity^8^. The results were analyzed using Generalize Additive Mixed Models (GAMM), with flexible treatment of random effect factors, as well as options for the modeling non-linear interactions of covariates (cf., Wood, 2006, 2008)^9^.

**Table 2:** Raw values of mean decision latency for targets after (form similar) semantically similar, dissimilar, and unrelated primes at five stimulus onset asynchronies (SOAs).

SOA	Prime type			Dissimilar vs. similar (difference in ms)
	Unrelated	Dissimilar	Similar	
34	680	667	661	6
48	696	690	660	30
67	701	681	653	29
84	698	672	660	12
100	699	682	674	8

#### Principal Component Analysis

A set of target attributes documented to be relevant in word recognition including log-transformed frequency, counts reported in the HAL study (Burgess and Livesay, 1998), log-transformed SUBTLEX frequency per million words (Brysbaert and New, 2009), word length (in characters), and form related neighborhood measures: number of orthographic neighbors (ON), number of phonological neighbors (PN), average distance to ONs (OLD20), and average distance to PNs (PLD20) were collected from the English Lexicon Project (Balota et al., 2007). Although each of these variables is useful to control, many of them are highly correlated. When they are included in analyses, this introduces a risk of multicollinearity, which was confirmed in the present study by a high condition number (κ = 49.94). To circumvent the problems associated wit h residualizing (see Wurm and Fisicaro, 2014), we applied a PCA, and then used PC scores as recombined and uncorrelated (orthogonal) predictors. Simply, principal component scores represent optimally weighted sums of the original set of variables with the goal of accounting for shared variance among related measures. An important feature is that they are particularly well suited for use in regression modeling (Dunteman, 1989).

**FIGURE 2:**
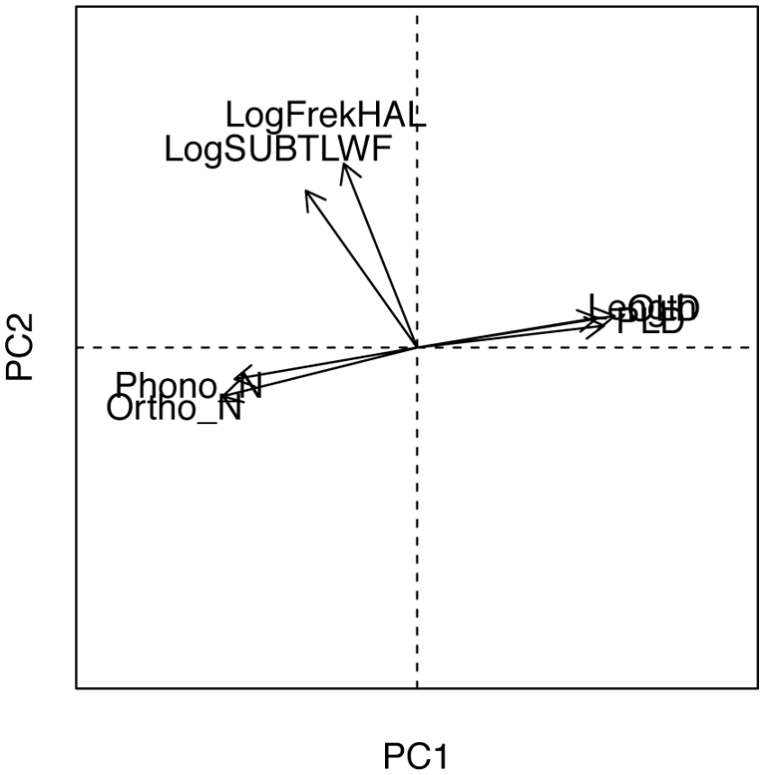
Vector representation of item variables in the plane defined by the form (PC1) and the frequency (PC2) principal components.

We kept only the first two PC components and their respective scores, as suggested by both the Kaiser–Guttman criterion (Horn, 1965) and the Scree-test (Cattell, 1966; Horn and Engstrom, 1979). These two components jointly explained about 77.5% of variance that the full set of the seven original predictors explained. **Figure 2** shows the biplot of the two extracted principal components. The first principal component (PC1) captures neighborhood properties. The length, OLD and PLD neighborhood measures have high positive loadings while the ON and PN counts have negative loadings. The interpretation of the PC1 is that a word with high positive score would be longer, would have fewer neighbors (taking into account negative loadings of ON and PN) and be at the greater distance from those neighbors (since OLD and PLD both have positive loadings). In summary, words that occupy large and dense orthographic and phonological neighborhoods have negative scores on the first component and should be easier to recognize. Conversely, words with negative scores on PC1 would be shorter, with many neighbors and at nearest proximity. In sum, words that occupy more scattered and less densely populated neighborhoods have positive scores on the first principal component and should end to be hard to recognize.

The second principal component (PC2) captures frequency-related variables: HAL frequency, and subtitle corpora frequency (SUBTLEX), both show very high positive loadings. Despite the fact that frequency, length, and various form-related neighborhood measures are highly collinear, with theoretically reasonable correlations (c.f., Zipf, 1935; Baayen, 2001), the PCA orthogonalization yielded a frequency dimension and a neighborhood dimension that were uncorrelated (i.e., orthogonal). We, thus, pursued statistical modeling with these uncorrelated principal components as our main continuous predictors – form and frequency covariates.

#### Generalized Additive Mixed Modeling: Five SOAs

In order to examine semantically similar and dissimilar primes so as to compare their time course of facilitation, the results from the two multiple SOA Experiments (1A,B) were jointly analyzed using a single generalized additive mixed effect model, retaining reciprocally transformed RT latencies as the dependent variable. We considered fixed effects of type of prime (semantically similar, semantically dissimilar, unrelated), SOA (34, 48, 67, 84, 100), and the interactions between these variables. SOA was defined as an ordered factor; hence, we were considering its linear and non-linear terms (quadratic, cubic, and fourth order; i.e., number of ordered levels minus one), both as a main effect and in interaction with the type of prime.

In addition to effects of SOA and prime type, the best model included additional non-linear effects called “smooth terms”: a tensor product of the two principal components and random effects for both target and prime word items and participant identity. The analysis also revealed that the frequency-related principal component (PC2) required additional by-participant adjustments for the slope. The final model was refitted and we removed those absolute standardized residuals exceeding 2.5. In this model *R*^2^ was 38%, on a final 8871 data points (after trimming). We describe the best model first and then elaborate on the contributions of smooth terms.

The primary analysis is reported in **Table 3**. It revealed that both prime type and its interaction with SOA as a linear term were statistically significant. The main effect of SOA, again a linear term, was also weakly significant (*p* = 0.04). The main effect of prime type indicated that responses after similar (i.e., transparent) primes were faster than after unrelated primes (β = -0.0834, *p* < 0.0001), and responses after dissimilar primes were faster than after unrelated primes (β = -0.0337, *p* = 0.0006). Further, by contrasting the two related types of pairs we confirmed that targets’ response latencies after semantically similar and dissimilar primes (SNEAKER-SNEAK vs. SNEAKY-SNEAK) were significantly different [Wald’s test: χ^2^(1) = 25.674; *p* < 0.0001]^10^.

**Table 3:** Generalized additive mixed model fitted to the lexical decision latencies for a range of SOAs (34, 50, 67, 84, 100), reporting parametric coefficients (A), and non-linear terms, tensor products, and random effects (B) with effective degrees of freedom (edf), reference degrees of freedom (Ref. df), *F* and *p*-values.

(A) Parametric coefficients	Estimate	SE	*t*-value	*p*-value
Intercept	-1.5482	0.0173	-89.3680	<0.0001
Prime type: dissimilar (S-)	-0.0337	0.0098	-3.4360	0.0006
Prime type: similar (S+)	-0.0834	0.0098	-8.4980	<0.0001
SOA linear (L)	0.0272	0.0132	2.0550	0.0399
SOA quadratic (Q)	-0.0089	0.0128	-0.7000	0.4841
SOA cubic (C)	0.0006	0.0263	0.0220	0.9822
SOA ˆ4 (4)	0.0056	0.0143	0.3930	0.6940
Prime type x SOA: S-× L	-0.0336	0.0149	-2.2510	0.0244
Prime type x SOA: S+ × L	-0.0600	0.0150	-4.0020	0.0001
Prime type x SOA: S-× Q	-0.0097	0.0153	-0.6380	0.5238
Prime type x SOA: S+ × Q	0.0225	0.0154	1.4650	0.1430
Prime type x SOA: S-× C	0.0257	0.0149	1.7210	0.0852
Prime type x SOA: S+ × C	0.0092	0.0150	0.6170	0.5375
Prime type x SOA: S-× 4	-0.0094	0.0156	-0.6060	0.5445
Prime type x SOA: S+ × 4	-0.0099	0.0157	-0.6340	0.5258

**(B) Smooth terms**	**edf**	**Ref. df**	***F*-value**	***p*-value**

TENSOR PRODUCT PC1 by PC2	7.2160	7.3460	5.6160	<0.0001
By-participant random intercepts	168.1320	176.0000	22.0310	<0.0001
By-participant random slopes for PC2	52.7390	177.0000	0.4380	0.0002
By-target random intercepts	37.0270	48.0000	15.2410	<0.0001
By-prime random intercepts	56.3350	150.0000	1.0760	0.0004

**FIGURE 3:**
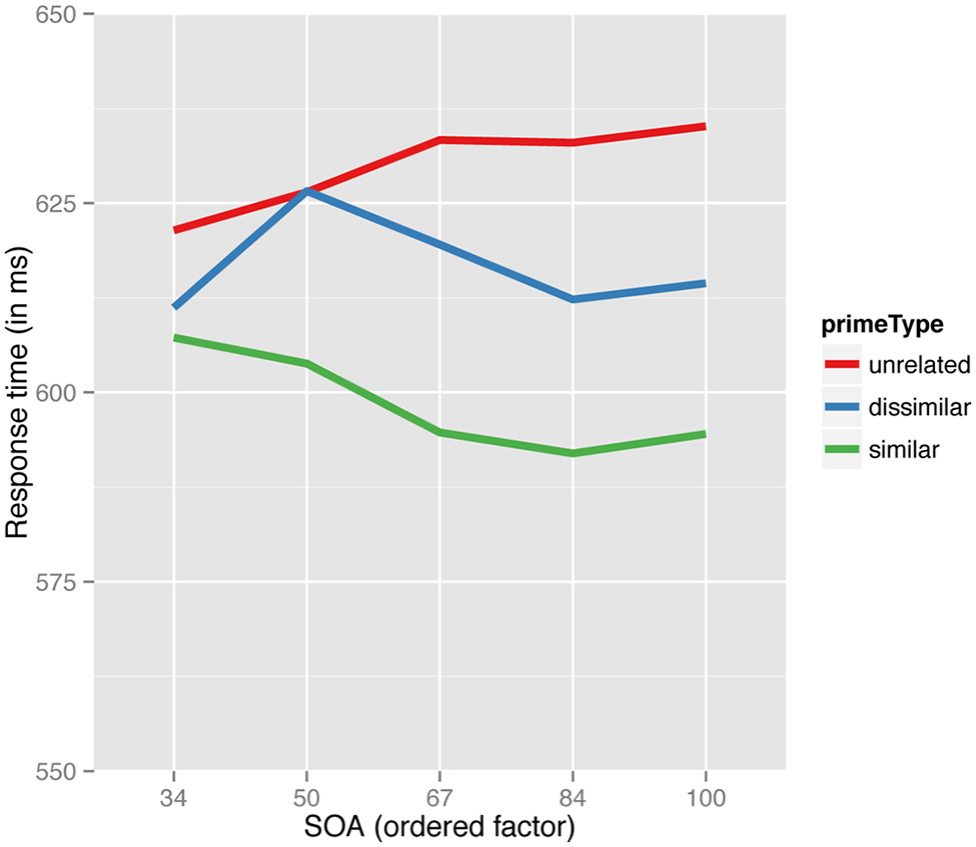
Predicted values for the partial effect of prime type by SOA (linear term) interaction for response times over a range of SOAs.

More interestingly, the linear term for rank-ordered SOA interacted with the type of prime. This interaction is depicted in **Figure 3**. For similar (transparent) pairs, as SOA increased, decision latencies decreased linearly then appeared to stabilize at the longest two SOAs (84 and 100 ms). Informative is that for the dissimilar (opaque) pairs we observed a weaker and later decrease in latencies as SOA increased. Specifically, a facilitation pattern is starting to emerge from the second shortest SOA (48 ms), rather than at the shortest SOA (34 ms) as in the case of similar (transparent) pairs. For targets after unrelated primes, (chalky- SNEAK) latencies slowly increase until the 67 ms SOA where they, become relatively stable. Although the analysis considered all linear, quadratic, cubic, and fourth order trends, only the linear trend reached significance. In the present study, we focus on whether the two coefficients of interest were statistically equivalent. That is whether there were differences in the linear trends over SOAs for dissimilar vs. similar primes. Wald’s test yielded a marginally significant difference [χ^2^(1) = 3.076, *p* = 0.08], suggesting that the decrease in latency for similar pairs is significantly steeper than for dissimilar pairs (see **Table 2**).

**FIGURE 4:**
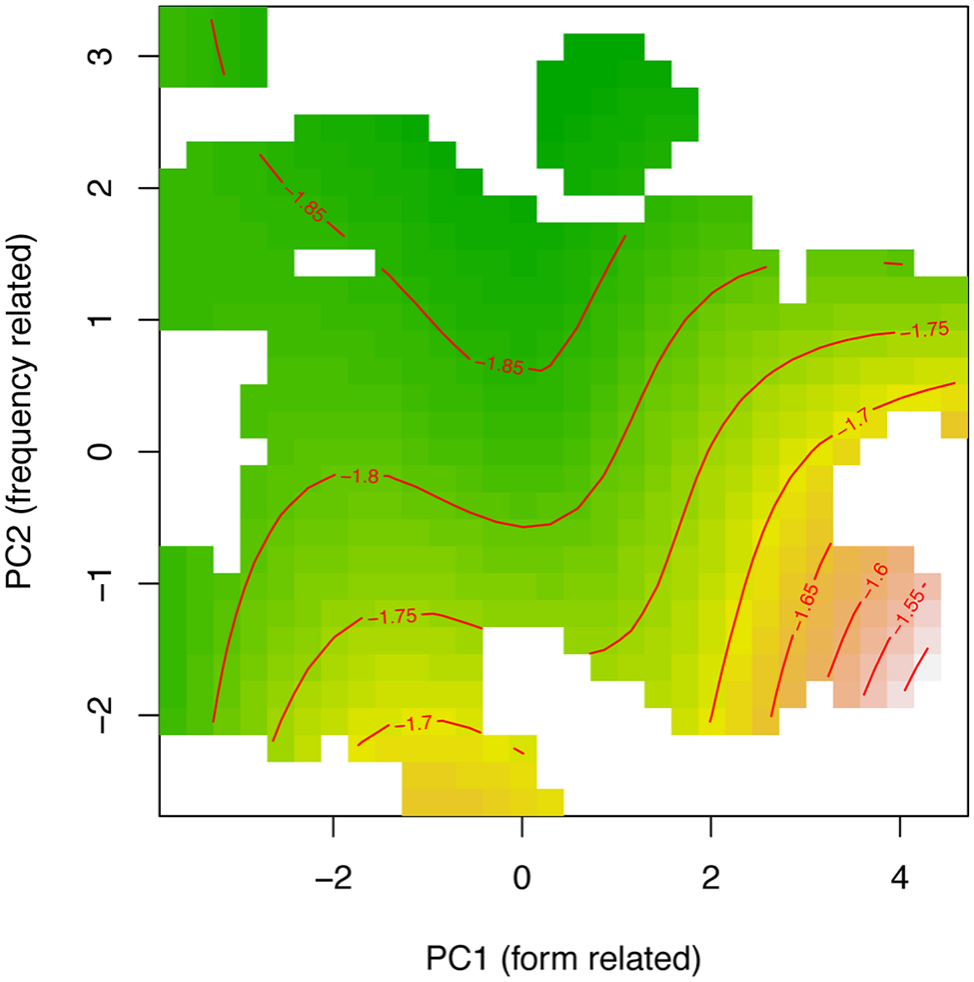
Tensor product smooths for the non-linear interaction of the principal component for form-related neighboring words (PC1) and frequency of occurrence (PC2). Green indicates shorter response latencies, and yellow-to-brown indicates longer response latencies at a range of SOAs ranging from 34 to 100 ms.

Smooth terms are listed in part B of **Table 3**. The first row of part B reports the non-linear interaction of PC1 and PC2. Including PCAs in the analyses accounts for much of the variability among targets. **Figure 4** shows the fitted surface projected on the PC1–PC2 plane, where shorter response latencies are presented with green and longer latencies are changing into yellow, orange, and then brown; Contour lines connect points on the surface that have the same latencies (that are the same height). This contour plot shows that response latencies tend to be long for words with large values on PC1 and low values on PC2. Simply stated, all else being equal, processing time increases for words that have fewer neighbors and are at a greater distance (positive values of PC1), especially when those words are low-frequency (negative values on PC2). The model also includes random intercepts for participants and items, both targets and primes. Finally, by-participant random slopes for PC2 also were statistically significant.

To summarize the analysis of target attributes, results show that a set of benchmark predictors, when reparameterized into two mutually independent principal components, entered into a strong non-linear interaction with decision latencies. In principle, the frequency-related PC2 effect is in the expected facilitatory direction (i.e., negatively correlated with latencies), although it is modulated by the characteristics of the target’s form-related neighborhood (PC1): words with few ONs, when scattered at a greater distance showed the most attenuated effect of frequency component (PC2). Several previous studies results have reported that neighborhood density facilitates decision time (c.f., Forster and Shen, 1996; Balota et al., 2004). In a study by Baayen et al. (2006), however, the neighborhood density effect disappeared when modeling allowed for a non-linear effect of word frequency. In the present study, therefore, we went a step further and tested for the interaction between the two composite predictors. The outcome demonstrates the interplay between a target’s neighborhood density and word frequency effects, and that recognition can benefit from both. Stated succinctly, words with low-density neighborhoods benefit least from their frequency of occurrence. Finally, the contribution of the frequency-related PC2 benefitted from an additional by-participant adjustment, meaning that the influence of frequency was modulated both, *generally*, by the target’s neighborhoods (number of neighbors and neighborhood density), and *specifically* by the differences between participants. This level of detail attests to the true complexity inherent to the dynamics of lexical processing and the excessive simplicity of models that treat all participants or all words as interchangeable.

#### Analyses Targeting Exclusively Short SOAs

The primary analysis tested for effects of prime type at five SOAs and included PCAs. In response to reviewer comments, we also report two additional analyses, one restricted only to derivations and a second only to the shorter SOAs. However, we wish to emphasize that the joint consideration of PCA and priming outcomes across all SOAs is preferable to separate analyses at each SOA because only the former model makes use of the full dataset and its power. Furthermore in the full analysis, we reported a significant interaction between SOA and prime type. One consequence of restricting the range of SOAs *post hoc*, is an increase in the chance of a type II error (H_0_ is false but accepted), basically because correlations tend to be attenuated by reduced variability (e.g., Sackett et al., 2007). Finally, partitioning the data with knowledge of the contents of the partitions, and then applying a statistical procedure designed as a test for random partitions is, by definition, selection bias – a known violation in statistics.

The raw means in **Table 2** deceive a reader into believing that there is no difference between dissimilar and similar facilitation at the shortest (34 ms) SOA^11^. However, the composite pattern across SOAs, participants and items reveals that the transparency effect is indeed present at 34 ms. Here, it is useful to remind the reader that our analysis of the time-course of facilitation treats SOA as a numerical – rank-ordered, rather than a nominal variable. This is important for two reasons. First, and most trivially, SOA is by its nature a numerical variable, and hence it should be treated as such; for instance, that 48 is bigger than 34 is an important component of the structure of the data, and this is wholly overlooked when analyzing multiple SOAs as unrelated nominal values. Second, and crucially, this enables us to exploit the power of non-linear regression to define the best-fitting line (or curve, if justified by the data) to account for the observed results^12^. To reiterate, if the critical interaction did significantly deviate from the linear trend that we observed in our analyses, it would have revealed itself in a higher order trend. Semantically similar pairs revealed no such interaction across the range of five SOAs.

Having professed to many concerns about the *post hoc* partitioning the data, at the request of reviewers, we examine the pattern of facilitation at SOAs of 67 ms and shorter to determine if the longer SOAs are responsible for the SOA by transparency interaction and the difference between prime types. In addition, we report analyses excluding the small number of compound primes so as to restrict prime-target pairs to derivations as did most of the previous studies on transparency.

Effects of semantic similarity with visible primes are incontrovertible and it is not impossible that some primes in some trials in the 84 and 100 ms SOA conditions were visible. Therefore here, we ask whether increases in the semantic similarity effect that we have documented with forward masked primes in Experiment 1 can be detected in the 34, 48, and 67 ms SOAs. Analysis showed that all significant effects in the analysis of the full dataset (five SOAs, from 34 to 100 ms) replicated almost perfectly. The only notable change was the weakening of facilitation for semantically dissimilar pairs. Most importantly, the difference between similar and dissimilar pairs across SOAs [5 SOAs χ^2^(1) = 3.076, *p* = 0.08] remained reliable at the three shortest SOAs [χ^2^(1) = 15.856, *p* = 0.001].

#### Analyses Targeted Exclusively at Derivations

As noted above, 8% (16/189) of the prime words were compounds rather than derivations. Therefore, to allay concerns that compounds could fabricate the early effect of semantic similarity between primes and targets, we ran two additional models: (a) removing only compound prime words (16 pairs) and (b) removing all targets that were paired with a compound in any of the prime conditions (nine targets with each of its three primes). In both analyses, similar to the previous *post hoc* analysis over the shortest SOAs (34–67 ms), the interaction of SOA with similar vs. dissimilar primes was robust [χ^2^(1) = 15.044, *p* = 0.0002].

It remains potentially informative to examine in more detail the pattern of facilitation at individual short SOAs because of claims that early processing relies on morpho-orthographic but not semantic properties of the prime, in which case differences between similar and dissimilar prime-target pairs (viz., semantic transparency effects) should not arise. That is our goal in Experiments 2 and 3, in each of which we concentrate power at a single SOA.

## Experiment 2

Across a range of five SOAs in Experiment 1, we observed that latencies to semantically related pairs decreased as SOA increases, with dissimilar pairs (opaque primes) showing this pattern later then similar pairs (transparent primes). In Experiment 2, we examine in more detail the pattern of facilitation at an SOA of 48 ms because these are the presentation conditions under which contention about early processing tapping not only into morpho-orthographic but also semantic properties of the prime has arisen (e.g., Amenta and Crepaldi, 2012). We continue to ask whether facilitation for semantically similar and dissimilar pairs differ. Further to determine whether that finding depends on exposure to a single vs. multiple prime durations, we compare the findings in Experiment 2 to those from the 48 ms SOA in the multiple SOA design of Experiment 1.

### Method

#### Participants

In Experiment 2 there were 84 participants from the same population as those in Experiment 1.

#### Materials, Design, Procedure

With the exception that all materials appeared at the single SOA of 48 ms, all dimensions were identical to Experiment 1. The items that were removed in Experiment 1 were again removed (PIG, FILL, SKIN, ABSENT, and SEED), for consistency.

#### Results and Discussion

**Table 4** summarizes means of prime type by single vs. multiple SOAs (i.e., Experiment 2 vs. the 48 ms SOA data from Experiment 1). As in our previous analysis, latencies on correct trials were transformed into negative reciprocals (-1000/RT) and we used principal component scores to include effects of frequency and form-related neighborhood density. Then data were submitted to analysis using GAMMs.

**Table 4:** Raw values of mean decision latency for targets after (form similar) semantically similar, dissimilar, and unrelated primes at 48 ms SOA, contrasting data from Experiment 1 with multiple SOAs, and Experiment 2 with 48 ms SOA only.

SOA	Prime type			Dissimilar vs. similar (difference in ms)
	Unrelated	Dissimilar	Similar	
Multiple (Experiments 1A, 1B)	696	690	660	30
Single (Experiment 2)	663	658	647	11

Fixed effects of type of prime (semantically similar, semantically dissimilar, unrelated), number of SOAs (single vs. multiple), and the interactions between these variables, together with scores on two principal components (form-related PC1, and frequency-related PC2), constituted the full set of predictor variables. Prime and target items and participants were random effect terms. **Table 5** reports the final model that was obtained after removing absolute standardized residuals larger then 2.5 units. The estimated explained variance of this model was *R*^2^ = 43%, on the remaining 11149 data points.

**Table 5:** Generalized additive mixed model fitted to the lexical decision latencies for 48 ms SOA, reporting parametric coefficients (**A**), and non-linear terms, tensor products, and random effects (**B**) with effective degrees of freedom (edf), reference degrees of freedom (Ref. df), *F*, and *p*-values.

(A) Parametric coefficients	Estimate	SE	*t*-value	*p*-value
Intercept	-1.554604	0.023844	-65.2	<0.0001
Prime type: dissimilar (S-)	-0.01604	0.008987	-1.785	0.0743
Prime type: similar (S+)	-0.058199	0.008993	-6.472	<0.0001
Number of SOAs: single	-0.058778	0.025126	-2.339	0.0193

**(B) Smooth terms**	**edf**	**Ref. df**	***F*-value**	***p*-value**

TENSOR PRODUCT PC1 by PC2	4.314	4.347	6.106	<0.0001
By-participant random intercepts	250.184	262	24.332	<0.0001
By-participant random slopes for PC2	43.971	263	0.209	0.0127
By-target random intercepts	41.319	51	35.542	<0.0001
By-prime random intercepts	62.024	153	1.52	0.0001

**FIGURE 5:**
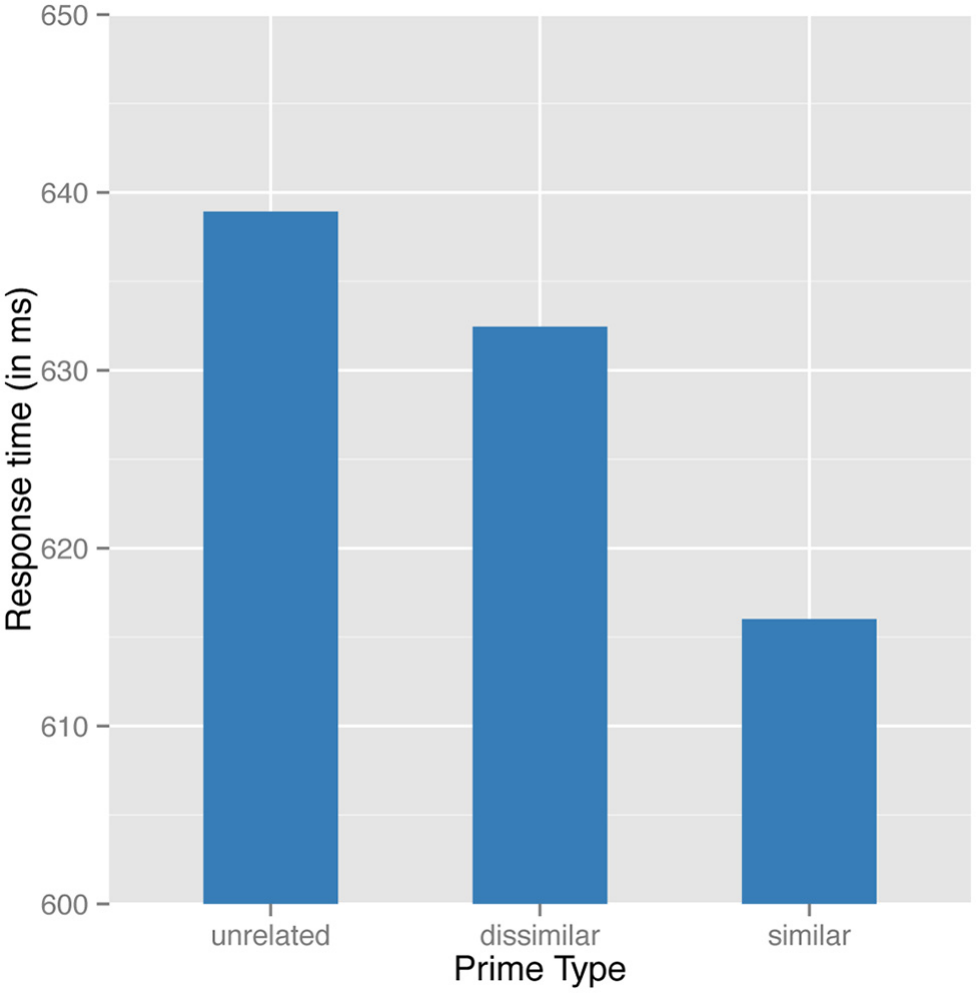
Predicted values for the partial effect of type of prime on decision latencies at a 48 ms SOA.

**Figure 5** represents the effect of prime type on response latencies at the 48 ms SOA. From **Table 5** we learn that similar pairs induced significant facilitation, compared not only to unrelated (β = -0.0582, *p* < 0.01 and β = -0.01604, *p* < 0.07, respectively) but also to dissimilar pairs [χ^2^(1) = 21.992; *p* < 0.0001]. At the same time, the difference between unrelated and dissimilar pairs was only marginally significant at the 48 ms SOA (β = -0.01604, *p* < 0.07, respectively; see **Figure 5**). We can conclude that transparency effects are robust and generalize across single and multiple SOAs. At the same time, differences between unrelated and dissimilar pairs at 48 ms SOA are more reliable when modeled from an experimental setting with a single SOA than with a range of SOAs.

**FIGURE 6:**
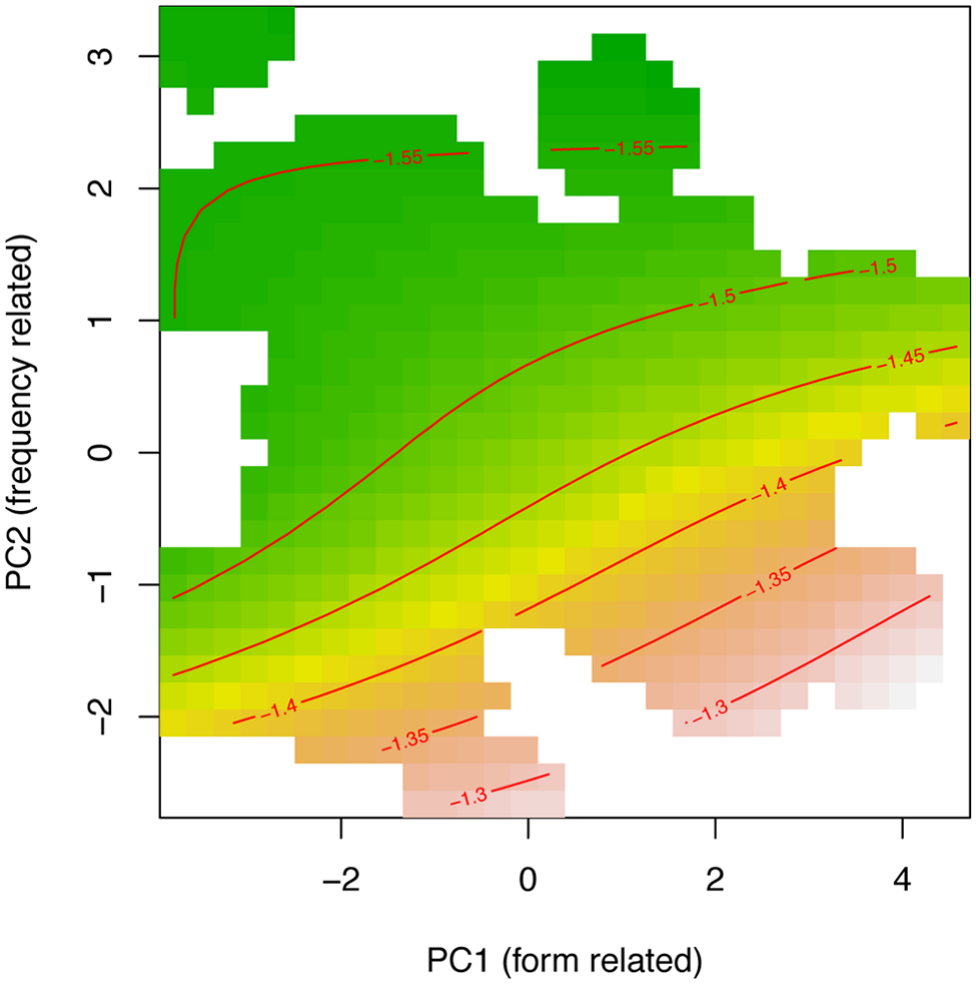
Tensor product smooth for the non-linear interaction of the principal component for form-related neighboring words (PC1) and frequency of occurrence (PC2). Green indicates shorter response latencies, and yellow-to-brown indicates longer response latencies at 48 ms SOA.

Finally, **Figure 6** shows a PC surface similar to the one in **Figure 4** (Experiment 1) for the 48 ms SOA when projected on the PC1–PC2 plane. In this case, the contour lines, that connect surface points with the same latencies, are slightly less wiggly and more stable. Nonetheless, the overall trends are quite comparable: longer response latencies for words with large values on PC1 and low values on PC2. As above, processing times are longer for low-frequency targets (PC2) with sparsely populated neighborhoods (PC1).

In sum, results based on the structure of the final model for the 48 ms SOA data support our claims based on the data collected over a range of SOAs. These include: (1) The main effect of the prime type: similar prime-target pairs contrast with both unrelated and dissimilar pairs, while the later two do not differ reliably. (2) The contribution of random effects, including the need for fine-tuning with by-participant slope adjustments for the frequency-related PC2. (3) A reliable non-linear interaction of the two principal components (PC1: neighborhood density; PC: frequency). (4) The effect of experimental setup with a single vs. multiple SOA contrast showing a small advantage for the pure 48 ms duration (*p* = 0.02). One point of modest divergence is that the difference between unrelated and dissimilar pairs at the 48 ms SOA is reliable with a single but not with multiple SOAs.

## Experiment 3

Experiment 1 revealed that the effect of semantic transparency was significant irrespective of SOA, and, additionally, that the difference between similar and dissimilar pairs increased, as SOA increased. Experiment 2 replicated the effect of semantic transparency at an SOA of 48 ms whether or not SOA varied during the course of the experimental session. Admittedly in Experiment 1, when one considered only those data points at the shortest SOA (34 ms), the advantage of transparency based on the contrast of semantically similar and dissimilar prime target pairs was statistically significant [Wald’s test: χ^2^(1) = 25.674; *p* < 0.0001], but not substantial (difference of 6.2 ms). Based on the 34 ms data from Experiment 1 alone, skeptics could argue, that the presence of a transparency effect at the shortest SOA is only an artifact of the advanced (and treacherously deceptive) modeling technique. At the same time, restricting the analysis only to responses at the 34 ms SOA would cause a dramatic reduction in experimental power, such that the null result would be minimally informative. In particular, if the effect at the 34 ms SOA is as small as predicted by the regression (on the order of less then 10 ms, see **Figure 3**), any reduction in power would make it almost impossible to observe the semantic effect in question.

In Experiment 3, we also probe for an interaction of reading skill and morphological processing. One previous study reported that fast readers show greater effects of letter transposition within (*vioilnist-VIOLINIST)* than between (*violiinst-VIOLINIST)* morphemes while slower readers do not (Duñabeitia et al., 2014). Even more relevant to the present study is the claim that differences between semantically similar and dissimilar pairs presented for 48 ms with a forward mask in the lexical decision task depend on a participant’s relative proficiency in spelling and vocabulary (Andrews and Lo, 2012, 2013). In addition to our basic design at a pure 34 ms SOA, in an attempt to ascertain influences of reading skill on morphological processing, we incorporated skills measures pertaining to vocabulary and spelling skill. In other respects, the prime-target materials and methods were identical to those in Experiment 2.

### Method

#### Participants

In Experiment 3 there were 73 participants from the same population as those in Experiments 1 and 2 who had not participated in either of the previous experiments.

#### Materials, Design, Procedure

With the exception that all materials appeared at the single SOA of 34 ms, the experimental setup was identical to Experiments 1. At the end of the experimental session all participants completed a spelling dictation and a vocabulary test.

#### Individual Difference Data

Two assessments of individual differences were introduced. The first was a spelling dictation test consisting of 15 items taken from Burt and Tate (2002). The second was a vocabulary 30-item vocabulary test taken from Andrews and Lo (2013). Each item was presented with five response options from which participants had to select the response that best defined the given word. Materials for the spelling dictation and vocabulary tests appear in Appendices B and C, respectively.

### Results and Discussion

#### Semantic Transparency

**FIGURE 7:**
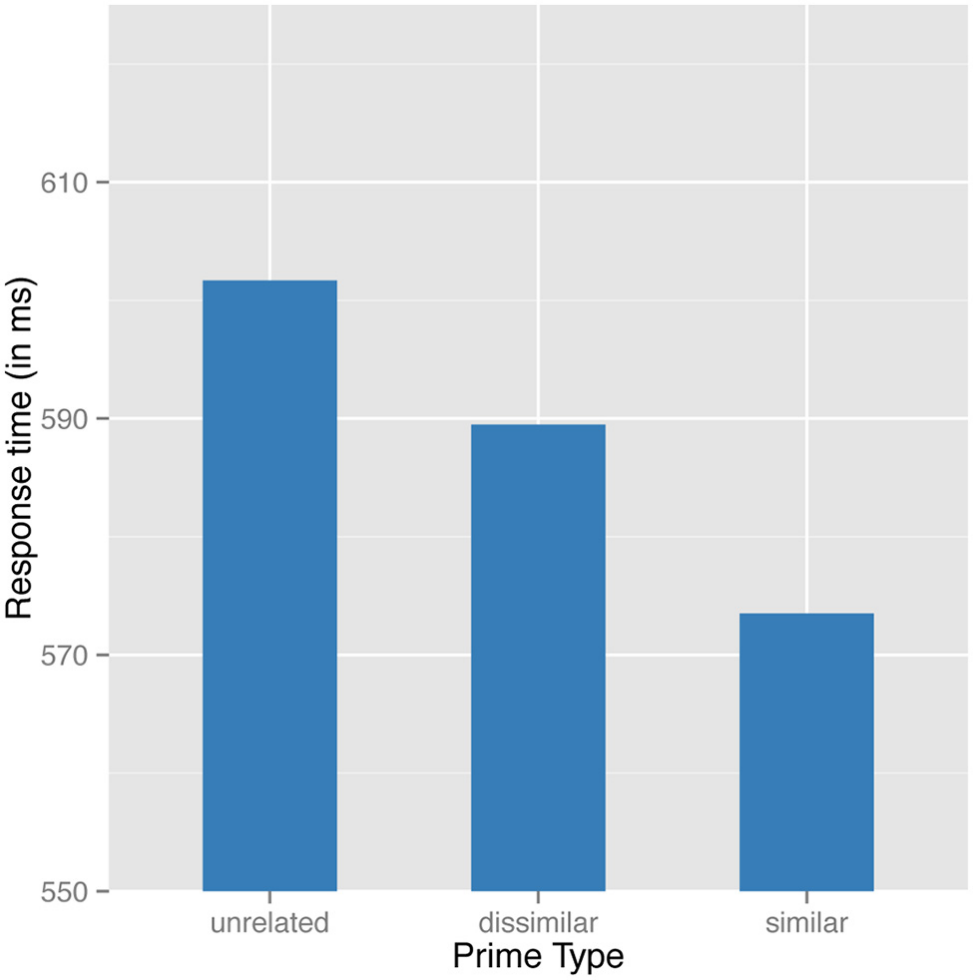
Predicted values for the partial effect of type of prime on decision latencies at a 34 ms SOA.

A generalized additive mixed effect model was fit to the reciprocally transformed correct RTs. This analysis revealed a main effect of prime type (with raw means for unrelated semantically dissimilar and similar primes of 652, 639, and 622 ms, respectively). Responses to semantically similar pairs, as well as to semantically dissimilar pairs, were significantly faster than to unrelated pairs (respectively: β = -0.0816, *p* < 0.0001; β = -0.0343, *p* < 0.002). Most crucially, similar pairs were significantly faster than dissimilar pairs [Wald’s test: χ^2^(1) = 19.605; *p* < 0.0001]. Notice that the present outcome replicates what was previously predicted by the model in Experiment 1 (compare **Figures 3** and **Figure 7**).

**FIGURE 8:**
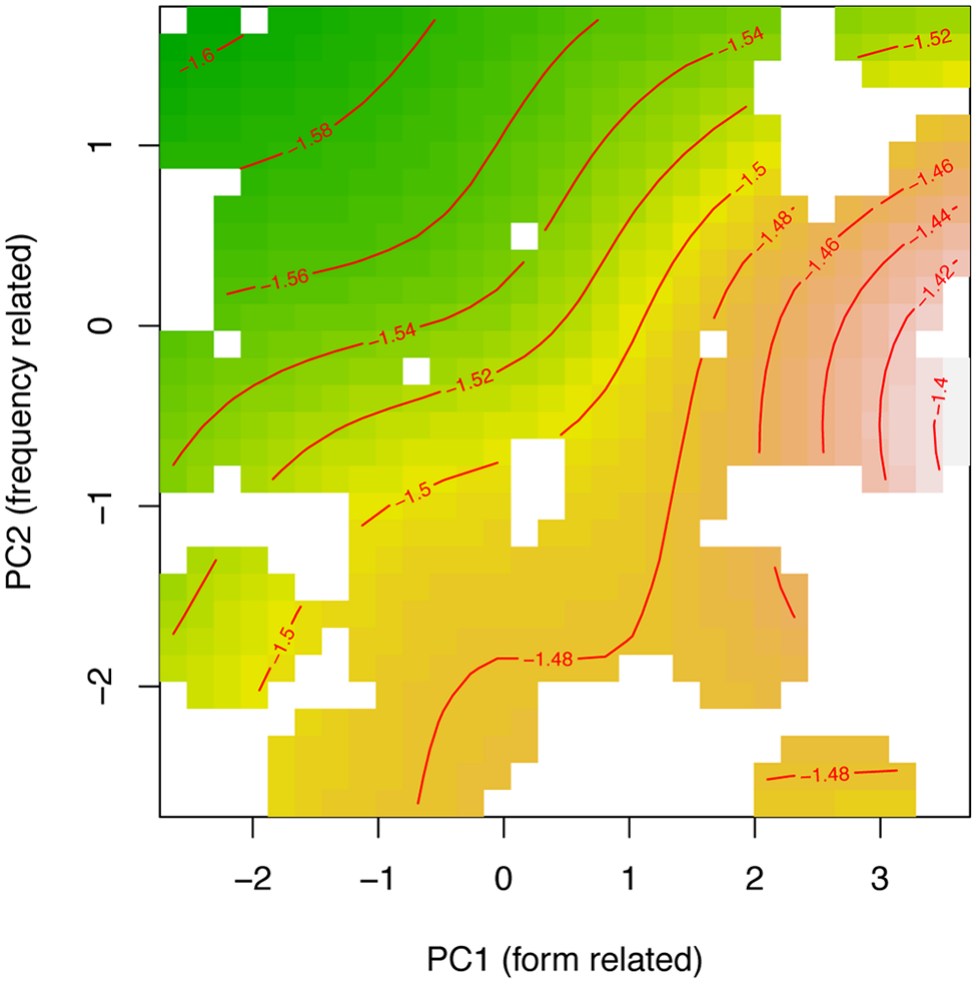
Tensor product smooths for the non-linear interaction of the principal component for form-related neighboring words (PC1) and frequency of occurrence (PC2). Green indicates shorter response latencies, and yellow-to-brown indicates longer response latencies at 34 ms SOA.

The tensor product in Experiment 3 appeared attenuated as compared with results from Experiments 1 and 2 (consult **Table 6** and **Figure 8**. Note that for the tensor product of PC1 by PC2, *p* ≈ 0.05). Additionally, the by-participant adjustment for the frequency-related PC2 was non-significant, as was the by-prime intercept adjustment. Overall, the model at 34 ms is simpler, although the main effect of prime type and the form and frequency PCs remained present. What does change is that the interaction between PC1 and PC2 was attenuated.

**Table 6:** Generalized additive mixed model fitted to the lexical decision latencies for 34 ms SOA, reporting parametric coefficients (**A**), and non-linear terms, tensor products, and random effects (**B**) with effective degrees of freedom (edf), reference degrees of freedom (Ref. df), *F*, and *p*-values.

(A) Parametric coefficients	Estimate	SE	*t*-value	*p*-value
Intercept	-1.6533	0.0399	-41.4390	<0.0001
Prime type: dissimilar (S-)	-0.0343	0.0107	-3.2060	0.0014
Prime type: similar (S+)	-0.0816	0.0107	-7.6450	<0.0001

**(B) Smooth terms**	**edf**	**Ref. df**	***F*-value**	***p*-value**

TENSOR PRODUCT PC1 by PC2	5.9570	6.1490	2.0880	0.0499
By-Participant factor smooths for Trial	245.1350	656.0000	4.7770	<0.0001
By-target random intercepts	39.2300	48.0000	6.6420	<0.0001
By-prime random intercepts	13.0360	150.0000	0.1000	0.1569

**FIGURE 9:**
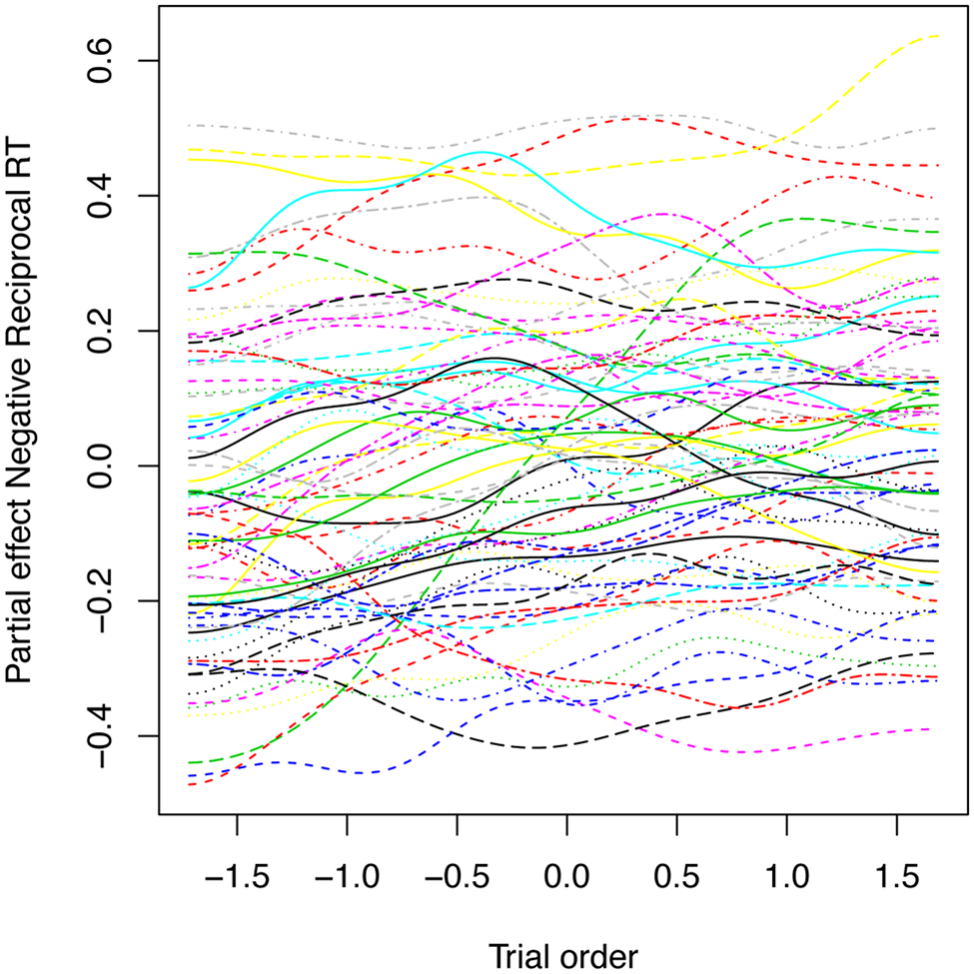
Partial effect of the by-participant random smooths for Trial in the generalized additive mixed model fitted to the primed lexical decision latencies with a 34 ms SOA. Each curve represents a different participant.

To probe for individual differences, we not only included two measures of reading skill (spelling proficiency and vocabulary), but also kept track of trial order, so as to maximize detection of individual variations. Thus trial order was entered into the model as a by-participant smooth factor for trials. It was highly significant (*F* = 4.777, *p* < 0.0001). **Figure 9** plots colored curves, one for each participant, representing how the participant’s performance changes over the course of the experiment. These changes can be attributed to numerous factors, such as learning, fatigue, and changes in attention.

Inclusion of by-participant factor smooths highlights the inter-trial dependencies in the response latency time-series. Incorporating both random by-participant adjustments for the intercept and, additionally, explicitly handling the response latency time-series (i.e., autocorrelation) per participant allows one to test for systematic individual differences that can be attributed to spelling proficiency and vocabulary.

#### Reading Proficiency and Morphological Processing

Rastle et al. (2004) claimed that early morphological processing is blind with respect to the semantic similarity of morphologically related prime and target, whereas Andrews and Lo (2012, 2013) claimed that readers who are more highly proficient in vocabulary than in spelling show effects of semantic similarity. In addition, Beyersmann et al. (2014) reported greater detrimental effects of partial morphological structure in non-words when proficiency was low. Nonetheless, Feldman et al. (2009; 2012) reported an effect of semantic similarity throughout their entire sample, regardless of reading skill. To explore the contribution of proficiency based on vocabulary and spelling dictation to effects of semantic transparency among morphologically related prime-target pairs, we compared four models that varied in their treatment of individual difference predictors.

The simplest model consisted of a dichotomized treatment of vocabulary (small vs. large) and spelling (low vs. high), similar to the methodology introduced by Andrews and Hersch (2010). To it we added our critical factor of prime type with three levels of prime-target relatedness (unrelated, dissimilar, and similar), and the random effect factor of target. Like Andrews and Hersch (2010), this model showed a strong main effect of dichotomized vocabulary (β = -0.0747, *p* < 0.0001), dichotomized spelling proficiency (β = -0.2212, *p* < 0.0001), and their interaction (β = 0.1916, *p* < 0.0001). The overall goodness of fit of this model, as expressed by Akaike’s Information Criterion was *AIC* = 2678.902.

A second model treated the two measures of individual differences in reading proficiency as continuous and possibly non-linear predictors and allowed for their interaction as well as including prime type and a random effect of target. This was a better model (*AIC* = 2202.487). The tensor product of vocabulary by spelling proficiency was highly significant (*edf* > 23, *F* = 30.008, *p* < 0.0001).

More interesting was the change that emerged when we entered the simplest possible term for a random effect of participants; namely, an intercept adjustment. This model (third in the sequence) showed a significant effect of prime type including the essential difference between semantically dissimilar and similar pairs (dissimilar: β = -0.0313, *p* < 0.006; similar: β = -0.0733, *p* < 0.0001), and significant random effects of both items (*edf* > 44, *F* = 7.202, *p* < 0.0001) and participants (*edf* > 64, *F* = 20.819, *p* < 0.0001). In this analysis and unlike Beyersmann et al. (2014), prime type failed to interact with the proficiency measures. In fact, the tensor product of vocabulary by spelling completely vanished (*edf* > 4, *F* = 1.241, *p* = 0.29) while the goodness of fit dramatically improved (*AIC* = 1355.909).

Finally, we introduced a by-participant factor smooth for trials. Of all models, this model achieved the best goodness of fit (*AIC* = 1162.259). At the same time, however, the tensor product of the two predictors of individual difference showed an increased *p*-value (*p* = 0.42), indicating their complete irrelevance for the model’s goodness-of-fit. Stated simply, the introduction of by-participant random variation over trials effectively outperformed our psychometric measures of individual differences in reading proficiency when predicting morphological processing and the role of early semantics.

As mentioned above, measures of reading proficiency should capture systematic differences between readers, whereas by-participant adjustments for the intercept and/or the factor smooths for trials are, by definition – random effects. Unfortunately, the proficiency measures we relied on failed to decant systematic from unsystematic participant-related co-determinants of word processing. The implication is that although individual differences are bringing new and exciting questions and answers to lexical processing and related fields, there is reason for caution. Psychometrics techniques can reveal robust indicators of systematic individual variations (e.g., Kuperman and Van Dyke, 2011; Van Dyke et al., 2014). They must be tested against unsystematic contributions such as behavioral variability during the course of the experiment, however.

#### Combined Analysis of Experiments 2 and 3

Ignoring the issue of selection bias described above, and complying with the request of reviewers, we combined the data from the 48 ms and the 34 ms SOAs into one analysis in order to further document early semantic effects. As in the separate analyses for each experiment, we used reciprocally transformed RTs as the dependent variable and considered prime type (unrelated, dissimilar and similar) and SOA (34 and 48 ms) as fixed factors along with the tensor product of PC1 and PC2 as a smooth term, and random effect of participants, primes and targets. In line with previous analyses, the final model also included significant by-participant adjustments for slope of the frequency- related PC2. All smooth terms were statistically significant.

Combined analysis of the data from Experiments 2 and 3 replicated the significant effect of prime type: both dissimilar and similar prime-target pairs had shorter response latencies than unrelated pairs (β = -0.0433, *p* < 0.0001 and β = -0.0796, *p* < 0.0001, respectively). Also consistent was the main difference between dissimilar and similar pairs [Wald’s test: χ^2^(1) = 10.844; *p* = 0.0001]. SOA (34 vs. 48) did not reach significance as a main effect (β = 0.0131, *p* = 0.6757), nor did the interaction of SOA with type of prime – i.e., increasing SOA did not alter the difference between each of the prime types (dissimilar: β = 0.0194, *p* = 0.0929; similar: β = 0.0211, *p* = 0.0678). Finally, with the two shortest values of SOAs for each of the two form related word pairs, values matched closely (β = 0.0194 vs. β = 0.0211) and tested statistically as indistinguishable [Wald’s test: χ^2^(1) = 0.021; *p* = 0.884].

## General Discussion

When the same targets were paired with semantically similar and dissimilar prime types, responses to semantically similar pairs were faster than to semantically dissimilar pairs and the latter differed only marginally from unrelated pairs. SOA in Experiment 1 was manipulated within an experimental block so as to enable us to track the time course of semantic contributions to morphological processing in a context where participants presumably apply the same processes to each trial. It could be argued that the presence of multiple SOAs within the same block, where some were consciously visible while others were only subliminal, induced strategic effects on lexical processing. However, a comparison of the data from the (pure) 48 ms SOA experiment with the 48 ms SOA data from the multiple SOA experiment failed to provide evidence for differences in the magnitude of facilitation. Instead, the only difference was that uncertainty as to when the target would appear in the multiple SOA experiment led to slower performance overall. To reiterate, multiple SOAs did not affect the magnitude of facilitation at a 48 ms SOA in any systematic way. The results of Experiment 3 confirm that the difference between semantically similar and semantically dissimilar morphologically related pairs was present and significant even at an SOA of 34 ms. In addition, the analysis combining the 34 and 48 ms SOA replicated the difference between semantically similar and dissimilar prime-target pairs. The difference increased between the SOAs of 34 and 48 ms too, but only marginally. Taken together, the model in **Figure 1B**, without a main effect of prime type, is not adequate.

Analysis of the short SOAs in Experiment 1 showed that the difference between semantically similar and dissimilar prime-target pairs increased with increasing SOA, whereas combining the 34 and 48 ms SOA data from Experiments 2 and 3 showed that the difference between semantically similar and dissimilar prime-target pairs was present in both and increased only marginally between the 34 and 48 ms SOA. We emphasize that the empirical contribution of our within experiment manipulation of SOAs is its potential to better depict the time-course over which formal and semantic contributions to morphological processing arise. Although several studies have contrasted semantic and morphological effects (Bentin and Feldman, 1990; Feldman and Soltano, 1999; Rastle et al., 2000, 2008; Feldman et al., 2004) and have reported that semantic contributions increase with SOA, to date details of the pattern have not been thoroughly delineated. In part, this is because different targets appeared with similar and dissimilar primes so that disparities among target sets could not be cleanly differentiated from priming effects. Further, prior studies considered only one or two SOAs in the range before priming transitions from subliminal to conscious. For example, Rastle et al. (2000; **Table 2**) reported a main effect of semantic transparency that appeared to increase between the 43 and 72 ms SOA, but magnitudes of facilitation for semantically similar morphologically structured pairs were atypically large (45–60 ms) and baselines after unrelated primes varied widely across target types and SOAs. These factors made it difficult to interpret increasing transparency effects with increasing SOA as fundamentally semantic rather than idiosyncratic to particular targets. Consequently from those data, one could not distinguish between the patterns represented in **Figures 1B–D**. The absence of detail was unfortunate given that the interaction of semantic transparency and SOA has become central to debates about models of morphological processing.

In this respect, a crucial innovation in the present study arises from considering SOA as the numerical variable that it naturally is. This enabled the explicit comparison of the multiple patterns of facilitation that correspond to different psychological theories. As we have seen, when the predictions of such theories are precisely described in the form of regression models, the results offer clear support for the model that is represented by a linear interaction. This method also offers a way of integrating the magnitudes of facilitation across SOAs. Furthermore, notice that the regression models have *predictive* value, one can interpolate and extrapolate the expected magnitudes of facilitation for other, not-observed, SOAs.

A model with a main effect of prime type as well as an interaction of prime type by SOA implies that early in the course of recognition there are contributions of both the formal and semantic aspects of morphological structure. Whereas the formal aspects behave less systematically at longer SOAs, semantic processing continues throughout a more extended period. This is consistent with a spread of activity across a word’s neural assembly as occurs in ‘full connectivity’ types of models, such as those of Pulvermüller (1999, 2001), Plaut and Gonnerman (2000), or Moscoso del Prado Martín (2007).

The form-with-meaning account contrasts with models that assume that the formal aspects of morphological processing, i.e., stem-affix parsing, must be completed before access to the semantic properties can succeed. Form then meaning models, such as that proposed by Rastle et al. (2004) and Rastle and Davis (2008), would predict a non-linear pattern more similar to that illustrated in **Figure 1D**.

In summary, counter to the claims from the morpho-orthographic segmentation account, regression analyses allow us to document effects of semantic similarity not only at 48 ms SOA but also at 34 ms SOA. While an effect of semantic transparency earlier than the 48 ms SOA might not be compelling from the means of Experiment 1 alone, they were fully reliable in GAMM and were replicated in Experiments 2 and 3. Thus, results failed to provide evidence for a qualitatively different and semantically blind style of processing at the earliest SOA.

### Reading Skill and Morphological Processing

Statistical predictors in complex models can obstruct each other’s contribution by competing to account for the same bits of variation in a dependent variable. We believe that a similar characterization applies to the two measures of individual differences in reading skill that we examined in Experiment 3. Stated bluntly, skill contributions vanished when we introduced other, stronger predictors based on random differences between participants. To garner support for this claim, we examined a series of models with progressively more complex treatments of spelling proficiency and vocabulary as predictors of morphological processing and evaluated each in terms of Akaike’s Information Criterion (AIC)^13^.

Our conclusion was that once the by-participant random variations were properly modeled, the psychometric measures of individual differences in reading proficiency contributed little to our understanding of morphological processing and the role of early semantics. Rather the proficiency measures that were expected to systematically influence patterns of morphological facilitation were almost certainly random (noise). This outcome highlights a general concern about the fashion of incorporating psychometric measures of individual differences to model experimental data and demonstrates the necessity to incorporate detailed model criticism as a default (e.g., Ramscar et al., 2014; Van Dyke et al., 2014).

### Is Morphological Similarity Without Semantics Really Morphological?

When primes are forward masked and presented for very short SOAs, some have differentiated between primes whose morphological structure is partially decomposable into morphemes and primes with fully decomposable morphological structure and have argued that only words with a fully decomposable morphological structure can facilitate their targets. To be more precise, targets (CORN) that follow partially decomposable primes (morpheme plus non-morphemic letter string like CORNEA (EA is not a morpheme) fail to differ from those that follow unrelated controls, whereas targets with exhaustively decomposable primes (all letter strings have the possibility to function as morphemes like CORNER that appears to be composed of CORN + ER) purportedly facilitate recognition of a target word. According to a morpho-orthographic account, facilitation arises only when the morphological structure of the prime allows exhaustive segmentation into possible morphemes (Rastle et al., 2004; Rastle and Davis, 2008). Recent results challenge the claim for a morphologically informed orthographic process by showing significant and equivalent facilitation after word primes that are partially and fully decomposable into morphemes (Milin et al., 2015) as well as after partially and fully decomposable non-word primes (Beyersmann et al., 2014). If both fully decomposable (affixed, pseudo affixed, and compound) words and partially decomposable words function comparably when primes are forward masked, then it becomes difficult to distinguish semantically dissimilar morphological from form-based processing.

In the present study we have demonstrated that even at an SOA of 34 ms, facilitation based on the appearance of a shared morpheme is weaker than facilitation based on semantic similarity in conjunction with a the appearance of a shared morpheme. Collectively, results call into question a rigid differentiation between morpho-orthographic and morpho-semantic stages of processes.

## Conclusion

The overall trend documented in the present study replicates both the findings and the meta-analysis of Feldman et al. (2009) in that when targets were held constant, semantically similar prime-target pairs produce greater facilitation than semantically dissimilar, form similar pairs. The unique contribution of the present study was to track the time course over which semantic factors influence recognition when primes are forward masked. Across five SOAs that varied from 34 to 100 ms, when random effects due to items and participants were controlled, the time course of facilitation varied for form-similar prime-target pairs with and without semantic similarity. Finally, semantic transparency effects were reliable even at a uniform 34 ms SOA. These findings replicate and extend the results of Feldman et al. (2009).

The opportunity to detect the linear increase of semantic transparency across SOAs underscores the value of concurrently treating subjects and items as random effects when analyzing latencies in repeated measures designs (Baayen et al., 2002, 2006, 2008; Forster and Masson, 2008). While an effect of semantic transparency in the earliest stage was not evident in the simple means of Experiment 1 that are reported in **Table 2**, consideration of the random effect structure and the systematicity of the relation between priming magnitudes and SOAs rendered this difference reliable in **Figure 2** based on a GAMM model. In fact in Experiment 3, the transparency effect was evident in the GAMM analysis even at an SOA of 34 ms. The outcome is consistent with a view of early lexical processing that entails extensive interaction between processes based on orthographic and semantic similarity.

Our data capture an early interaction of meaning with form. This interaction is noteworthy because it is inconsistent with a characterization of visual word recognition as a sequence of independent (morpho-orthographic then morpho-semantic) components or processes and highlights, instead, the dynamics of their interaction. Developments in cognitive neuroscience likewise are shifting away from an emphasis on independent brain regions and their function toward less localized networks with the potential for complex interactions at multiple scales. The interaction of semantics with form processing whose time course we have tracked may be representative of a style of processing in which traditionally conceived later processes influence purportedly earlier ones. With few exceptions (e.g., Plaut and Gonnerman, 2000; Moscoso del Prado Martín, 2007) models of visual word recognition typically posit independent and sometimes rate varying semantic and orthographic processes. A benefit early in the course of processing from semantic similarity between a morphological stem in isolation and in a morphologically complex prime word context is not easy to reconcile with models of word recognition that stipulate complete form analysis before analysis of meaning can begin. The outcome suggests that form and meaning properties of words or their constituents can be processed concurrently or otherwise influence each other. In essence, it challenges the universality of the form-then-meaning assumption within models of word recognition.

## Conflict of Interest Statement

The authors declare that the research was conducted in the absence of any commercial or financial relationships that could be construed as a potential conflict of interest.

## Footnotes

1 We eschew the terms transparent and opaque because while semantically dissimilar pairs like SNEAKER-SNEAK are true morphological relatives, that is not the case for pairs like CORNER-CORN. The morpho-orthographic accounts treats them as comparable even though there is no morpheme internal to CORNER whose semantics can be evaluated for transparency.

2 We use the term “potential” because ER can be an affix (e.g., FARMER) but is not in the context of CORNER.

3 The Feldman and Soltano study used unmasked SOAs of 48 and 250 ms.

4 Extrapolation will be enhanced as well.

5 The processing of compound words is subject to very similar debates about transparency as is morphological derivation (de Jong et al., 2000, 2002; Moscoso del Prado Martín et al., 2004a,b, 2005; Kuperman et al., 2008; Baayen et al., 2011). Specifically, transparency effects have been documented with compounds as well as morphological derivations and further, transparency can moderate effects of whole word and constituent frequency (Kuperman et al., 2009). Nonetheless, the claim that early processing is morpho-orthographic but semantically blind typically applies to patterns of facilitation after decomposition of derivations and generally ignores compounds. The most obvious justification is that in derivations, stems are privileged components when they are segmented from the morphemes with which they combine whereas the privileged status of one morpheme over another is less plausible in the context of unaffixed compounds because its two or more components contribute more equally to the meaning of the morphologically complex word. Stated generally, derivations and compounds behave more similarly when components are examined in relation to each other and appear most dissimilar when individual non-stem components are removed and stems are inspected in isolation.

6 Under forward masked conditions in our experience, ON primes that fail to preserve the first letter with the target fail to produce facilitation. Therefore, occasional single letter overlap of an unrelated prime with the target is unlikely to alter the baseline relative to an unrelated prime that shares so letters.

7 Rating data appear in the Appendix.

8 Box Cox transformations of the power function (Box and Cox, 1964) revealed that reciprocal transformations maximized normality for the data. Additionally, we used a negative reciprocal with base of 1000: -1000/RT, as advised by Baayen and Milin (2010). This way, with negative values we preserve all effects in expected direction, and with a 1000 base wider, more appropriate range of transformed values.

9 We did not consider applying logistic additive mixed effect models to the error date due to very high accuracy, with fewer than 5% errors.

10 Modeling techniques standardly use a reference level for categorical predictor (i.e., factor) to set the intercept, and then form comparison(s) for the remaining level(s) of that predictor. Thus, if there are more then two levels, non-referent levels are not directly compared. One statistically sound way to test for a specific difference is to use a test like Wald’s. It tests the null hypothesis that a set of parameters is equal to some specified value (for details consult Fox and Weisberg, 2011). If the test does not reject the null hypothesis, this suggests that removing the parameters (which numerically define variables) from the model essentially would not harm model fit. For example, if a model has a three-level factor one will be selected as a reference and the differences to other two will be tested appropriately. It is possible, however, that the Wald’s test between the other two, non-referencing levels, come out as non-significant. Then, it would be justified to pool those two levels together and to simplify the model that will, then, have only one comparison: the one between the reference level and the combined levels, which have been tested as insignificantly different.

11 The ordering of latencies for means in **Table 2** indicates that semantically similar (SNEAKY-SNEAK) pairs were recognized faster than dissimilar pairs (SNEAKER-SNEAK), which in turn were faster than unrelated pairs (CHALKY-SNEAK). Considering only the mean RTs, this pattern is clearly visible at the intermediate but less evident at the shortest SOA. As explained above, we caution that these means are deceptive because they are not adjusted for systematic variability due to participant, target, prime or SOA, even though the experiments were designed with the deliberate goal of treating SOA as a numerical. Thus, we base our conclusion about differences between primes types as a function of semantic transparency on the more powerful modeling technique across SOAs, rather than on a comparison between arithmetic means in **Table 2**.

12 Any apparent lack of a non-linear effect based on the means could be a simple consequence of noise. However, even if one did attribute the absence of a non-linear effect to noise, that would not be sufficient to claim that a non-linearity is present so as to be able to claim that early processing is semantically blind and that semantic effects appear only later (**Figure 1D**).

13 We point out that in our sample, the association between facilitation and reading and spelling skill, around which Andrews and Lo (2013) built their analyses and discussion, was absent. It is only fair to note, however, that our spelling measure entailed only dictation whereas theirs also included other measures. However, it is likely that types of spelling tests and the interrelation are not the real issue. Andrews and Lo (2013) handled the high intercorrelation between two standardized measures of individual differences of written language proficiency (ZSpell and ZVocab) by applying a principal component analysis (PCA). With this approach, in essence, the authors reparametrized the original variables into mutually independent (or orthogonal) predictors. This approach might be preferable to the more typical use of residuals of collinear predictors, but it is certainly not without problems (Wurm and Fisicaro, 2014). Crucially, PCA runs its component extraction in a sequential and greedy fashion; i.e., components are obtained one-by-one, each forced so as to explain a maximum of the unexplained variance, given the original set of variables. This “first served rule” grossly favors “first borns,” leaving only “screes” for the remaining components (for further discussions about screes and the number of *true* principal components consult seminal works by Cattell (1966) and Horn and Engstrom (1979). Crucially, this is the situation in Lo and Andrews’ PCA: the first component captured 84% of the common variance, and the second component acquired the remaining 16%. In other words, with two variables that were subjected to PCA, the total variance is 2 (in standardized units where each of the initial variables has variance 1). The consequence is that in the analysis by Lo and Andrews, the second principal component explains only 32% of the variance of any of the two initial variables (ZSpell or ZVocab). The rest is consumed by the first principal component. Having exceptionally high positive correlations (i.e., loadings) of the first principal component on the one hand, and the original spelling and vocabulary measures on the other hand, one cannot distinguish between the individual contributions of the two language proficiency measures in the linear modeling that ensues. For the reason enumerated above, in the present study we deliberately pursued a different methodology. In particular, we did not submit our original variables of written language proficiency to a PCA. The correlation between the two variables in the present case was relatively low (*r* = 0.35). Thus, the issue of collinearity was not urgent. Furthermore, we utilized only two measures of individual differences of written language proficiency and that allowed for explicit testing of their respective contributions as predictors when modeling reaction time latencies (this manner of handling of predictors in wide-range linear modeling has its own advantages; see Wurm and Fisicaro, 2014). Ultimately, we tested the two predictors by gradually tightening the specification of random effects related to variations across participants. In this way, progressively and explicitly and in a conservative manner, we have tested whether two measures of language proficiency could serve as valid indicators of “systematic” differences between individuals.
