# Supplementary material for: Must analysis of meaning follow analysis of form? A time course analysis
Source: Front Hum Neurosci. 2015 Mar 11;9:111. doi: 10.3389/fnhum.2015.00111 (PMC4366802; doi:10.3389/fnhum.2015.00111)
Supplement: Supplementary file 1 [file data_sheet_1.pdf]

Supplementary Materials for: Must analysis of meaning follow analysis of form? A time course analysis

Laurie Beth Feldman[1,2], Petar Milin[3,4], Kit Cho[1], Fermín Moscoso del Prado Martín[5] and Patrick A. O'Connor[1]

[1] University at Albany, State University of New York

[2] Haskins Laboratories

[3] Eberhard Karls University Tübingen, Germany

[4] University of Novi Sad, Serbia

[5] University of California, Santa Barbara

Appendix A: Materials

Target	Sem Similar Prime	Sim rating	Sem Dissimilar Prime	Dissim rating	S-D	Unrelated
ABSENT	absently		absentee			Detainee
ARCH	archway	6.2	archer	3.6	2.6	Driveway
AUDIT	auditor	5.5	auditorium	2.4	3.1	Traitor
BAT	batter	4.4	battery	2.5	1.9	Flicker
BOMB	bombard	3.7	bombastic	3.2	0.6	Gymnastic
BRAND	branded	4.8	brandy	2.2	2.6	Beady
BUZZ	buzzer	6.1	buzzard	3.1	3.0	Unitard
CAB	cabstand	3.4	cabbage	1.6	1.9	Headstand
CANDID	candidly	5.7	candidate	2.2	3.5	Validate
CAVE	cavern	5.2	cavity	3.0	2.3	Acidity
CELL	cellule	3.6	celery	1.6	2.0	Fishery
COAST	coastal	6.2	coaster	3.3	3.0	Growler
CODE	codify	4.6	codeine	1.8	2.8	Typify
CORD	cordless	4.0	cordial	2.3	1.8	Denial
COUNT	countable	5.5	county	2.6	3.0	Adorable
CRATE	crated	4.6	crater	2.8	1.8	Booted
CURE	curable	6.2	curator	2.9	3.3	Aviator
CUSTOM	customary	5.7	customer	2.2	3.5	Rotary
CUTE	cuteness	6.7	cuticle	1.5	5.3	Icicle
DENT	dented	6.3	dentist	1.6	4.7	Flutist
DIVIDE	divisor	4.8	divisive	4.1	0.7	Elector
DOLL	dolly	4.8	dollar	1.7	3.1	Pulsar
EAR	earplug	5.3	earmark	3.1	2.2	Sparkplug
EARN	earnable	5.5	earnest	3.3	2.2	Amenable
FAIR	fairness	6.3	fairy	1.9	4.4	Vastness
FILL	filling	5.9	filly	3.6	2.3	Purring
FLAT	flatly	5.7	flatware	3.3	2.4	Meekly
FLOAT	flotation	6.0	flotilla	2.3	3.7	Gorilla
FRUIT	fruity	5.9	fruitless	3.7	2.2	Bulky
GENE	genetic	6.3	genial	3.5	2.9	Facial
GLOSS	glossy	6.1	glossary	2.1	4.0	Urinary
HOST	hostess	6.4	hostage	2.0	4.5	Poetess
IRON	ironware	4.4	irony	2.1	2.3	Hardware
LAB	labor	2.8	labial	2.0	0.7	Partial
LIQUID	liquify	5.5	liquidate	4.1	1.4	Fixate
MESS	messy	6.7	messmate	3.4	3.3	Bunkmate
PALACE	palatial	3.1	palatable	1.7	1.4	Notable
PATH	pathway	6.6	pathology	2.6	4.0	Mythology
PIG	piglet	6.4	pigment	1.7	4.8	Triplet
PINK	pinkest	5.4	pinkie	2.8	2.6	Keenest
PROOF	proven	5.9	proofing	5.0	0.9	Toughen

PURE	purity	6.4	puree	2.8	3.6	Goatee
QUOTE	quotation	6.4	quotient	2.4	4.0	Coalition
RAT	ratty	3.7	ratify	1.5	2.1	Fortify
RELY	reliable	6.0	relish	1.8	4.2	Variable
SAUCE	saucy	5.5	saucer	3.0	2.5	Banker
SAVE	savings	6.0	savant	2.5	3.5	Defiant
SEED	seedless		seedy			Endless
SKIN	skinless	3.9	skinny	3.1	0.8	Beefy
SLIME	slimy	6.1	slimmer	2.1	4.0	Oily
SNEAK	sneaky	6.4	sneaker	2.5	3.9	Chalky
SOFT	soften	6.3	softball	2.9	3.4	Deafen
SORE	soreness	6.5	sorely	4.4	2.2	Tidiness
STAB	stabber	6.0	stabile	2.1	3.9	Missile
STATUE	statuette	4.5	statuary	3.5	1.0	Canary
STORE	storage	4.8	storybook	2.1	2.7	Checkbook
STRIDE	striding	5.9	strident	3.2	2.7	Feigning
STRING	stringy	5.9	stringer	3.8	2.2	Bugler
SWEAT	sweaty	6.5	sweater	3.1	3.3	Mighty
TART	tartness	5.4	tartar	3.0	2.4	Illness
THICK	thicken	5.6	thicket	3.3	2.3	Anklet
TREAT	treatment	6.0	treaty	2.4	3.6	Pavement
WEAR	wearable	5.6	weary	2.4	3.2	Filmable

Appendix B:
Materials for the spelling test

accomplice
broccoli
decadent
euphoric
filament
graffiti
gullible
impotent
lingerie
minstrel
pinnacle
ravenous
scenario
succinct
vigilant

Appendix C:
Materials for the vocabulary test

1. Toxin
a. poison
b. weed
c. failure
d. belief
e. disease

2. Competition
a. achievement
b. capacity
c. repayment
d. selection
e. rivalry

3. Symptom
a. harmony
b. outline
c. indication
d. result
e. invention

4. Partition
a. spasm
b. passageway
c. division
d. bias
e. treatment

5. Annul
a. approve
b. enforce
c. revise
d. corrupt
e. cancel

6. Retard
a. resume
b. delay
c. substitute
d. announce
e. break

7. Sever
a. label
b. learn
c. scold
d. mend
e. cut

8. Chronicle
a. mural
b. history
c. clock
d. large pimple
e. jar

9. Adept
a. actual
b. extreme
c. skilled
d. distinct
e. fortunate

10. Imaginary
a. insulting
b. fancied
c. exaggerated
d. foolish
e. temporary

11. Lore
a. knowledge
b. interval
c. sermon
d. crack
e. cuff

12. Concord
a. victory
b. harmony
c. structure
d. danger
e. weight

13. Supple
a. condensed
b. shrewd
c. flexible
d. useful
e. cheerful

14. Valid
a. sound
b. brave
c. moral
d. careful
e. neutral

15. Refute
a. recover
b. seek
c. enroll
d. control
e. disprove

16. Adorn
a. paint
b. praise
c. fasten
d. ornament
e. admire

17. Turbulent
a. bulging
b. towering
c. agitated
d. powerful
e. concealed

18. Allege
a. assert
b. warn
c. suspect
d. lie
e. decide

19. Gibe
a. injure
b. gossip
c. scoff
d. point
e. doubt

20. Gaudy
a. happy
b. frozen
c. showy
d. worthless
e. clumsy

21. Edifice
a. building
b. body of laws
c. design
d. assistance
e. entrance

22. Sinuous
a. polished
b. dangerous
c. dark
d. feminine
e. winding

23. Undue
a. unready
b. exposed
c. unexpected
d. excessive
e. scanty

24. Cacophony
a. adjunct
b. link
c. officer
d. gesture
e. discord

25. Pallet
a. rug
b. fence

c. garden
d. trough
e. bed

26. Evince
a. report
b. display
c. evade
d. develop
e. detect

27. Censure
a. blame
b. suppress
c. deny
d. register
e. resolve

28. Incursion
a. sudden invasion
b. condemnation
c. spiral movement
d. repulse
e. investigation

29. Choreography
a. the art of singing
b. the art of carving
c. the art of painting
d. the art of dancing
e. the art of weaving

30. Divagation
a. undersea exploration
b. location of position
c. wandering
d. observation of stars
e. separation
